# The Armadillo (*Dasypus novemcinctus*): A Witness but Not a Functional Example for the Emergence of the Butyrophilin 3/Vγ9Vδ2 System in Placental Mammals

**DOI:** 10.3389/fimmu.2018.00265

**Published:** 2018-02-23

**Authors:** Alina Suzann Fichtner, Mohindar Murugesh Karunakaran, Lisa Starick, Richard W. Truman, Thomas Herrmann

**Affiliations:** ^1^Institut für Virologie und Immunbiologie, Julius-Maximilians-Universität Würzburg, Würzburg, Germany; ^2^National Hansen’s Disease Program, Louisiana State University, Baton Rouge, LA, United States

**Keywords:** Vγ9Vδ2, TRGV9, TRDV2, butyrophilin 3, coevolution, nine-banded armadillo, placental mammals

## Abstract

1–5% of human blood T cells are Vγ9Vδ2 T cells whose T cell receptor (TCR) contain a *TRGV9/TRGJP* rearrangement and a *TRDV2* comprising Vδ2-chain. They respond to phosphoantigens (PAgs) like isopentenyl pyrophosphate or (E)-4-hydroxy-3-methyl-but-2-enyl-pyrophosphate (HMBPP) in a butyrophilin 3 (BTN3)-dependent manner and may contribute to the control of mycobacterial infections. These cells were thought to be restricted to primates, but we demonstrated by analysis of genomic databases that *TRGV9, TRDV2*, and *BTN3* genes coevolved and emerged together with placental mammals. Furthermore, we identified alpaca (*Vicugna pacos*) as species with typical Vγ9Vδ2 TCR rearrangements and currently aim to directly identify Vγ9Vδ2 T cells and BTN3. Other candidates to study this coevolution are the bottlenose dolphin (*Tursiops truncatus*) and the nine-banded armadillo (*Dasypus novemcinctus*) with genomic sequences encoding open reading frames for *TRGV9, TRDV2*, and the extracellular part of *BTN3*. Dolphins have been shown to express Vγ9- and Vδ2-like TCR chains and possess a predicted *BTN3*-like gene homologous to human *BTN3A3*. The other candidate, the armadillo, is of medical interest since it serves as a natural reservoir for *Mycobacterium leprae*. In this study, we analyzed the armadillo genome and found evidence for multiple non-functional *BTN3* genes including genomic context which closely resembles the organization of the human, alpaca, and dolphin *BTN3A3* loci. However, no *BTN3* transcript could be detected in armadillo cDNA. Additionally, attempts to identify a functional *TRGV9/TRGJP* rearrangement *via* PCR failed. In contrast, complete *TRDV2* gene segments preferentially rearranged with a *TRDJ4* homolog were cloned and co-expressed with a human Vγ9-chain in murine hybridoma cells. These cells could be stimulated by immobilized anti-mouse CD3 antibody but not with human RAJI-RT1B^l^ cells and HMBPP. So far, the lack of expression of *TRGV9* rearrangements and *BTN3* renders the armadillo an unlikely candidate species for PAg-reactive Vγ9Vδ2 T cells. This is in line with the postulated coevolution of the three genes, where occurrence of Vγ9Vδ2 TCRs coincides with a functional BTN3 molecule.

## Introduction

With up to 5% of T cells, Vγ9Vδ2 T cells constitute a major γδ T cell population in the human blood (1, 2). Their T cell receptor (TCR) is characterized by a pairing of a Vγ9 chain, encoded by a *TRGV9/TRGJP* gene rearrangement and a *TRGC1* constant region, and a Vδ2 chain using a *TRDV2* variable region. This cell subset recognizes and rapidly reacts to endogenous or exogenous phosphoantigens (PAgs) in a MHC-unrestricted fashion (1). PAgs are small molecules with pyrophosphate groups produced during isoprenoid synthesis. The most important naturally occurring PAgs are isopentenyl pyrophosphate and (E)-4-hydroxy-3-methyl-but-2-enyl pyrophosphate (HMBPP). The importance of the Vγ9Vδ2 T cell subset lies within their multitude of effector functions such as production of cytokines, killing of cells (*via* TCR, NKG2D, CD16), B cell help and APC-like functions (2). Their reactivity to aminobisphosphonates and PAgs makes them a potential tool for tumor treatment (3) and involvement in infections with HMBPP-producing pathogens like *Mycobacterium tuberculosis* (4–8), *Mycobacterium leprae* (9), *Listeria monocytogenes* (10) and in malaria (11) and toxoplasmosis (12) was observed. The implication of Vγ9Vδ2 T cells in infections has been reviewed elsewhere (13, 14). Recently, Butyrophilin 3 (BTN3) (CD277) has been proven essential for the PAg-dependent activation of Vγ9Vδ2 T cells (15). The three human BTN3 isoforms belong to the immunoglobulin superfamily and their expression has been shown on T and B cells, monocytes, NK cells, dendritic cells (16–18), and non-hematopoietic cells (19). In humans and other primates, the *BTN3* gene was subject to two successive duplications resulting in three isoforms BTN3A1, A2, and A3 (20). These share the same overall structure: two extracellular immunoglobulin-like domains (BTN3-V and BTN3-C) and a transmembrane region. The isoforms BTN3A1 and A3 additionally possess an intracellular B30.2 domain, which is missing in BTN3A2 (21). Regarding Vγ9Vδ2 T cells, BTN3A1 seems to mediate PAg recognition through the B30.2 domain containing a positively charged surface pocket, which can accommodate PAgs (15). The molecule BTN3A1, however, is not sufficient to induce PAg-mediated Vγ9Vδ2 T cell activation and other unknown molecules on the human chromosome 6 are currently investigated (22).

The long-standing belief that Vγ9Vδ2 T cells are a primate-specific T cell subset has lately been challenged through studies in other placental mammals. Genomic surveys demonstrated the existence of *TRGV9, TRDV2*, and *BTN3* genes in several species of placental mammals but not in other mammals or vertebrates (23, 24). Therefore, an emergence of those genes with Placentalia seems evident. The best candidate for a non-primate species bearing PAg-reactive γδ T cells is, so far, the alpaca (*Vicugna pacos*), which possesses transcripts of γδ TCR rearrangements with features typical of human PAg-reactive cells (23) and transcripts of a BTN3 ortholog with high homology to primate BTN3. In line with this, our group generated first evidence for PAg-reactive γδ T cells in this species (25).

Apart from that, the bottlenose dolphin (*Tursiops truncatus*) has recently been found to express *TRGV9*- and *TRDV2*-like productive rearrangements (26) and a *BTN3A3*-like gene was predicted *via* Gnomon gene prediction tool (GenBank: XM_004332447.2). Another candidate with in-frame *TRGV9, TRDV2*, and *BTN3* extracellular domain genes is the nine-banded armadillo (*Dasypus novemcinctus*), which belongs to the Xenarthra superorder. Armadillos are a natural reservoir of *M. leprae* and, therefore, a valuable tool for leprosy research (27, 28). In addition, the neurological involvement and dissemination in armadillos infected with *M. leprae* is similar to the one observed in humans and could not be reproduced in rodent models, as reviewed elsewhere (29). Karunakaran et al. (23) predicted armadillo *TRGV9* and *TRDV2* genes with rather high identities to their human homologs as well as a *BTN3-V*-like domain. In this study, we tested the expression of those genes in armadillo PBMCs. Here, we report the expression of *in silico* translatable *TRDV2* chains but the apparent lack of expression for productive *TRGV9* rearrangements and of a complete *BTN3*-like transcript and discuss the implications of these findings for the coevolution of *Vγ9, Vδ2*, and *BTN3* genes.

## Materials and Methods

### Armadillo/Alpaca/Dolphin Homologs for *TRGV9, TRDV2*, and *BTN3*

*Dasypus novemcinctus* (taxid 9361) whole genomic shotgun sequences (wgs) were taken from the National Center for Biotechnology Information (NCBI) databases (BioProject: PRJNA12594/PRJNA196486; BioSample: SAMN02953623; GenBank: gb|AAGV00000000.3). Homologous sequences to human Vγ9Vδ2 TCR MOP (GenBank: KC170727.1/KC196073.1) or G115 (PDB: 1HXM_A) (30) and BTN3A1/2/3 (GenBank: NM_007048.5/NM_007047.4/NM_006994.4) were predicted using the NCBI Basic Local Alignment Tool (BLAST) (31). Accession numbers of identified armadillo homologs are: *TRGV9* AAGV03121505.1 nt402-695; *TRGC-A* Ex1 AAGV03121543.1 nt3646-3947; *TRGC-B* Ex1 AAGV03121550.1 nt3170-3471; *TRGC-C* Ex1 AAGV03121548.1 nt6289-6590; *TRGC-D* Ex1 AAGV03173223.1 nt672-373; *TRDV2* AAGV03208792.1 nt2277-1994; *TRDC* Ex1/2 AAGV03208782.1 nt782-510/nt95-27; *TRDC* Ex3 AAGV03208781.1 nt 1291-1218; 1st *BTN3-V-ID* AAGV03145787.1; 2nd *BTN3-V* AAGV03287843.1; 3rd *BTN3-V* AAGV03240336.1; 2nd *BTN3-C* AAGV03240337.1; 3rd *BTN3-C* AAGV03010207.1.

*Vicugna pacos* (taxid 30538) whole genomic shotgun sequences were obtained from NCBI databases (BioProject: PRJNA30567, BioSample: SAMN01096418). A full-length alpaca BTN3-like sequence amplified from *V. pacos* cDNA (MG029164) (32) and an alpaca *BTN3* gene predicted by NCBI via Gnomon (XM_015251744.1) were used to analyze the genomic organization of the alpaca *BTN3* locus in the contig ABRR02153549.1.

*Tursiops truncatus* (taxid 9739) wgs sequences were obtained from NCBI databases (BioProject: PRJNA356464 and PRJNA20367, BioSample: SAMN06114300 and SAMN00000070) and two loci with *BTN3*-like genomic regions were found (*BTN3-V-ID* MRVK01002630.1 and *BTN3-V-C* ABRN02485746.1). A predicted *BTN3*-like molecule (XM_004332447.2) was used for BLAST analysis of wgs data.

Gene regions in *BTN3* loci were assigned according to consensus splice donor and acceptor sites. If no consensus splice site was found, the exon length was determined *via* homologies to human *BTN3A3* exons. If not otherwise indicated, the IMGT nomenclature was used for *TRG* and *TRD* genes and transcripts from human and mouse and if possible, armadillo genes were named according to their homologies to human genes. If not, letters were used to indicate different isoforms. The proteins encoded by *TRGV9* and *TRDV2* rearrangements are referred to as Vγ9 and Vδ2 TCR chains, respectively.

### Amplification of Armadillo *TRGV9, TRDV2* Rearrangements, and *BTN3* Transcripts

Armadillo PBMCs in RNAlater and genomic liver DNA were provided by the National Hansen’s Disease Program, Baton Rouge, LA, USA. Armadillos were maintained and samples collected in accordance with all ethical guidelines of the U.S. Public Health Service under protocols approved by the IACUC of the National Hansen’s Disease Program, assurance number A3032-1.

RNA isolation was performed with RNeasy Mini Kit (Qiagen) and First Strand cDNA Synthesis (Thermo Fisher Scientific) was performed with Oligo dT primer after DNase digestion with DNase I (Thermo Fisher Scientific). Unknown 5′ and 3′ ends of transcripts were determined using the GeneRacer Kit with SuperScript III RT (Invitrogen) according to the manufacturer’s instructions. Touchdown PCR with RACE-ready cDNA was performed with Q5 Hot Start Polymerase (NEB) and Phusion Polymerase (Thermo Fisher Scientific) was used for other PCR experiments. TOPO TA cloning set for sequencing with pCR4-TOPO vector (Thermo Fisher Scientific) was used for cloning and sequencing of PCR products. Armadillo genomic liver DNA was used as a control for PCR amplifications. Primer sequences are given in Supplementary Table S1 in Supplementary Material.

#### TRDV2

*TRDV2*/*TRDC* amplification was performed with the primers A21 and A72, nested PCR with A71 and A73. The 5′ end of *TRDV2* was determined *via* 5′RACE PCR with the primer A118 and nested primer A119. The primers A94 and A95 were applied for 3′RACE PCR starting from *TRDV2*. The PCR products of those amplifications were subsequently cloned and clones were analyzed.

#### TRGV9

Attempts to amplify a *TRGV9* rearrangement included amplification of *TRGV9*/*TRGC* with different primer combinations and 3′RACE PCR starting from *TRGV9*. The 5′ end of *TRGC* transcripts was, therefore, amplified using 5′RACE PCR and the primers A86 and A87, and the PCR product was cloned with the TOPO TA cloning kit. The 3′ sequence of *TRGC* was analyzed with 3′RACE PCR using the primers A103 and A104.

#### Butyrophilin 3

Expression of a *BTN3* homolog in armadillo PBMCs was analyzed with the partial amplification of *BTN3* from the *BTN3*-*V* to *BTN3-C* domain with primers specific for all three armadillo homologs (A122 + A123). Furthermore, RACE PCR to obtain the 5′ sequence of *BTN3-V* (A165, A166) and the 3′ sequence from *BTN3-V* (A163, A164) and *BTN3-C* (A167, A168) was conducted.

### Sequence Analysis

Sequence analysis of genomic sequence data or PCR amplifications was performed with NCBI BLAST and Clustal Omega software. Alignments were calculated with Clustal Omega and BioEdit software was used for editing of alignments.

### Expression of Armadillo Vδ2 TCR Chains

A murine TCR-negative T cell hybridoma cell line (BW58 r/mCD28) expressing a rat/mouse chimeric CD28 molecule (33, 34) was used to express armadillo Vδ2 TCR chains and test for surface expression, CD3 signaling, and HMBPP-reactivity. Full-length armadillo Vδ2 chains were amplified using the primers A193 and A194 and cloning in pMSCV-IRES-mCherry FP (a gift from Dario Vignali, Addgene plasmid # 52114) was performed using the In-Fusion^®^ HD Cloning Kit (Takara Bio). The clones 7 and 9 were selected for co-expression with the human Vγ9 TCR MOP chain (35). Retroviral transduction of BW58 r/mCD28 cells was used to stably express TCR chains (36) and vector-encoded EGFP (pEGN huVγ9) and mCherry (pMSCV dnVδ2 cl7 or cl9) indicated successful transduction. TCR surface expression was confirmed in a flow cytometry staining of human Vγ9 (2.5 µg/ml anti-Vγ9 TCR 4D7 mAb) (37) detected by a secondary antibody [1 μg/ml F(ab')2 Fragment Donkey α-Mouse IgG (H + L)] (BD Pharmingen) and anti-mouse CD3 (1 µg/ml biotin hamster anti-mouse CD3ε clone 145-2C11) detected by streptavidin [0.4 µg/ml Streptavidin-APC (BD Pharmingen)]. BW58 r/m CD28 cells overexpressing transduced TCR chains can be applied as responder cell lines in various *in vitro* models of antigen recognition and their activity can be measured by mouse IL-2 ELISA (38, 39). Thus, the human/armadillo TCR transductants (hu/dnTCR cl7 or cl9) were tested for functional TCR signaling by CD3 crosslinking and PAg reactivity (HMBPP, Sigma-Aldrich) in co-culture with Raji RT1B^1^ cells (23, 38, 40). TCR-negative BW58 cells expressing r/mCD28 (TCR^–^), the same cells transduced with only the human Vγ9 chain (hu/-TCR), and the human TCR MOP (hu/huTCR) were used as controls for stainings and stimulations. Cells were cultured in 200 μl/well RPMI 1640 supplemented with 5 or 10% FCS, 100 mM sodium pyruvate, 0.05% w/v glutamine, 10 mM nonessential amino acids, and 5 × 10^−5^ M mercaptoethanol (Invitrogen). Stimulations were carried out for 22 h with 5 × 10^4^/well responder cells cultured in 96-well round bottom plates (Greiner) in co-culture with 5 × 10^4^/well RAJI-RT1B^1^ cells. For CD3 crosslinking, 96 well flat bottom plates (Greiner) were coated with anti-mouse CD3ε (clone 145-2C11, BD Pharmingen) in PBS for 24 h at 4°C before stimulations. Mouse IL-2 sandwich ELISA (BD) was used to determine IL-2 secretion in the culture supernatants and appropriate dilutions were measured if the upper detection limit was reached.

## Results

### Genomic Organization of a Close Homolog of Human *BTN3* loci in Armadillo

**Figure 1:**
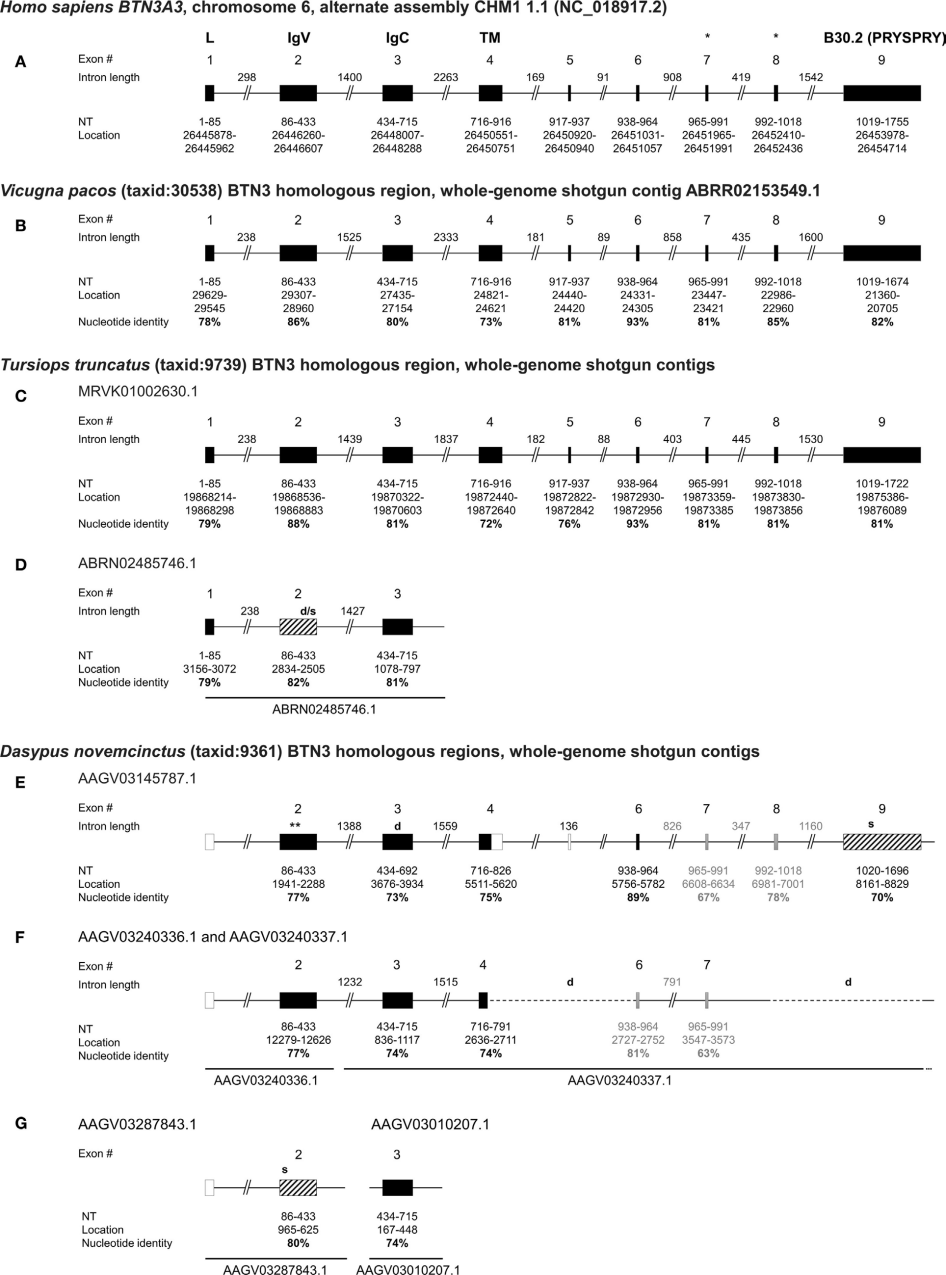
Genomic organization of armadillo Butyrophilin 3 (*BTN3*) homologous regions *BTN3-V, BTN3-C*, and B30.2 show similarities to human, alpaca, and dolphin *BTN3* loci. The human *BTN3A3* locus **(A)** was determined by National Center for Biotechnology Information (NCBI) megablast of BTN3A3 (GenBank: NM_006994.4) to Human G + T database (GenBank/Assembly: NC_018917.2). The alpaca *BTN3*-like locus **(B)** was mapped using NCBI blastn of the predicted alpaca *BTN3A3* (XM_015251744.1) to *Vicugna pacos* wgs database. Dolphin *BTN3*-like loci **(C,D)** were identified using NCBI blastn of the predicted dolphin *BTN3A3* (XM_004332447.2) to *Tursiops truncatus* wgs. Armadillo *BTN3* homologous regions **(E–G)** were identified by NCBI blastn of human BTN3A3 to *Dasypus novemcinctus* whole genome shotgun contigs database (taxid: 9361). Exons are represented by boxes: translatable (solid black), non-translatable (striped black), missing (solid white) found by intron homologies (solid gray). The size of the exon, location in assembly/contig, and nucleotide identity of the regions to human *BTN3A3* are indicated in bold. Intron lengths were calculated based on location in contigs and putative deletions are shown by dashed lines and “d”. Stop codons are indicated by “s” at the approximate location in the gene (*location of the proposed juxtamembrane motif important for PAg recognition (41); **location of the putative ATG at nt 1982).

Previous studies reported armadillo genomic regions homologous to the human *BTN3A1* extracellular and intracellular domains (24). After more detailed homology analysis of those armadillo genes, a closer resemblance to human *BTN3A3* was confirmed. Through the NCBI Basic local alignment (BLAST) tool (31), we, therefore, compared the human *BTN3A3* mRNA sequence (GenBank: NM_006994.4) to the *D. novemcinctus* whole genomic shotgun sequences (wgs) and could identify three homologous regions for the *BTN3*-V and *BTN3*-C domains, respectively. To compare these with homologous *BTN3* genes in other species, we additionally analyzed the *BTN3*-like loci of the two other candidate species alpaca (*V. pacos*) and bottlenose dolphin (*Tursiops truncatus*). For those species, predicted *BTN3A3*-like sequences are published in NCBI databases (alpaca: XM_015251744.1, dolphin: XM_004332447.2). Those predicted sequences were compared to the respective wgs databases to analyze *BTN3*-like loci and isoforms. Whole genome shotgun sequence databases are comprised of contigs with unique accession numbers and contain incomplete non-annotated genomic information. Whole genomic shotgun sequences were taken from the NCBI databases and allow full or partial reconstruction of *BTN3* encoding genomic regions (Figure 1). The corresponding nucleotide and amino acid sequence alignments and armadillo locus information are supplied in the Figures S1–S5 in Supplementary Material.

The human *BTN3A3* gene is comprised of nine protein-coding exons with exon 2 encoding the BTN3-V region, exon 3 encoding BTN3-C, exon 4 representing part of the transmembrane region, followed by four relatively small exons (5–8) and the B30.2 exon (9) (Figure 1A) (42). The alpaca *BTN3*-like genomic sequence is organized in a locus strikingly homologous to human *BTN3A3* (Figure 1B), showing exons with nucleotide sequence identities to human *BTN3A3* ranging from 73 to 93% and conserved intron lengths. The intracellular B30.2 domain is slightly shorter (81 nt) than the human counterpart. *In silico* splicing and translation of this alpaca *BTN3*-like gene with an overall nucleotide identity of 81% to human *BTN3A3* results in a protein sequence, which shares 72% amino acids with the human homolog. The expression of an alpaca BTN3-like molecule (GenBank: MG029164), with a conservation of 81% on the nucleotide and 73% on the amino acid level to human BTN3A3, has been confirmed before (32). We could, however, identify minor differences between the genomic alpaca *BTN3* and the *BTN3* transcript amplified from cDNA on the nucleotide and amino acid level (Figures S1 and S2 in Supplementary Material). This can be explained by interindividual polymorphisms that also exist in humans (20). Both available alpaca BTN3 protein sequences carry six conserved amino acids each in the BTN3-V (Glu37, Lys39, Arg61, Tyr100, Gln102, and Tyr107) and B30.2 domain (His351, His378, Lys393, Arg412, Arg418, Arg469) (Figure S2 in Supplementary Material) predicted to be involved in PAg recognition in human BTN3A1 (15, 23, 24, 43).

Dolphins have been found to express *TRGV9*- and *TRDV2*-like mRNA transcripts (26), however, *BTN3* expression has not yet been proven. Here, we report the existence of one locus in the dolphin wgs database that comprises a full-length *BTN3*-like sequence predicted by NCBI *via* Gnomon (GenBank: XM_004332447.2) and a remarkably conserved locus organization (Figure 1C). Comparable to the alpaca *BTN3*-like locus, the dolphin locus features nine exons with nucleotide (nt) identities from 72 to 93% compared to human *BTN3A3* and intron lengths similar to the one in the human *BTN3A3* locus. However, the intron between exon 6 and 7 is only about half in size compared to the human intron at this location and the intracellular B30.2 exon (9) is 33 nucleotides shorter. The dolphin *BTN3*-like sequence *is in silico* translatable and exhibits a nucleotide identity of 81% and an amino acid (aa) identity of 73% with human *BTN3A3* (Figure S1 and S2 in Supplementary Material). This *BTN3A3*-like gene carries five out of six conserved amino acids in the BTN3-V domain and a substitution (Lys39Thr) (Figure S2 in Supplementary Material). All six predicted PAg-binding residues in the B30.2 domain (15, 43) are identical. Interestingly, we report the existence of another *BTN3*-like partial locus in the dolphin genomic sequences (Figure 1D). This contig is only long enough to comprise exons 1 to 3 of a *BTN3*-like gene structure. The *BTN3*-V (exon 2) of this locus (Figure 1D) is 92% identical to and shorter than the other *BTN3*-V found for the dolphin (Figure 1C), which indicates possible deletions in this exon. Consequently, this locus seems to code for a *BTN3*-like pseudogene.

Database analysis of the armadillo wgs database resulted in a total of three *BTN3*-V, three *BTN3*-C homologous regions, and one exon similar to the human *BTN3A3* B30.2 domain. One pair of *BTN3-V* and *BTN3-C* is comprised in one single contig of the nine-banded armadillo wgs database (AAGV03145787.1), which also includes a partial hit for the transmembrane region in exon 4 of *BTN3A3*, three small exons, homologous to human exons 6–8, and a downstream B30.2-like region (Figure 1E; Figure S5A in Supplementary Material). All those homologous regions show a nucleotide conservation of more than 70% compared to human *BTN3A3* domains and are also remarkably similar to human *BTN3A3* with respect to intron lengths and genomic organization. Two other *BTN3-V* domains (AAGV03287843.1 and AAGV03240336.1) were found as well as two other *BTN3-C* domains (AAGV03240337.1 and AAGV03010207.1). However, the *BTN3-C* containing contig AAGV03240337.1 does not seem to include a B30.2-like region and shows a truncated transmembrane homolog directly followed by another exon similar to the transmembrane region of human *BTN3A3* (Figure 1F; Figure S5B in Supplementary Material). No homologous intracellular regions could be found in AAGV03010207.1 due to the short contig length (Figure 1G). Owing to the abundant use of SPRY/B30.2 domains in several families of molecules (44), prediction of *BTN3*-related B30.2 regions is difficult, except for the one found in contig AAGV03145787.1 (Figure 1E; Figure S4 in Supplementary Material). Additionally, conserved leader sequences encoded by exon 1 and another part of human *BTN3A3* encoded by exon 5 could not be predicted in all contigs through Blast using *BTN3A3* and it is noteworthy that gene prediction tools like Gnomon used for a predicted armadillo *BTN3A3* entry (GenBank: XM_012528284.1) or FGENESH^+^ (reference protein: huBTN3A1/2/3; GenBank: NM_007048.5/NM_007047.4/NM_006994.4) (45) also fail to predict a leader sequence in the AAGV03145787.1 contig. The published predicted *BTN3A3* homolog calculated by Gnomon software and our own calculations with FGENESH^+^ locate the start codon within the *BTN3-V* region (Figure 1E). *In silico* translation was successful for two *BTN3-V*-like regions (Figure S3A in Supplementary Material). The third *BTN3-V* homolog in AAGV03287843.1 (Figure 1G) carries a stop codon, if translated in the same frame. All three *BTN3-C* homologs were translatable; however, the respective region in AAGV03145787.1, although not having any stop codons, exhibits one nucleotide deletion leading to a frameshift (Figure 1E; Figure S3B in Supplementary Material). The only intracellular B30.2 domain found in this setting in the armadillo is identical with the previously reported one (24), but reexamination of the nucleotide to protein translation reveals several stop codons if the human B30.2 frame is used (Figure S4 in Supplementary Material). Yet, nucleotide alignments show the conservation of codons encoding all of the six conserved PAg-binding residues in the B30.2 domain of BTN3A1 described by Sandstrom et al. (15) including His351. Six extracellular PAg-binding residues have been proposed for the BTN3-V domain of BTN3A1 (43) and codons for these amino acids are partially conserved in the armadillo. Here, four out of six codons are conserved in the *BTN3*-V exons found in AAGV03240336.1 and AAGV03287843.1, and three out of six in AAGV03145787.1.

In addition to database analysis, we tested for expression of potential *BTN3* isoforms, as well as *TRGV9* and *TRDV2* transcripts, in cDNA of armadillo PBMCs. *D. novemcinctus* PBMCs dissolved in RNAlater were provided by the National Hansen’s Disease Program, Baton Rouge, LA, USA and tested for transcripts of *BTN3, TRGV9*, and *TRDV2*. These PCR approaches included the amplifications of *BTN3* performed with primers specific for all *BTN3*-V and *BTN3*-C regions and the RACE PCR amplification of the 5′ and 3′ sequences starting in several domains of the predicted genes (Table S1 in Supplementary Material). No transcripts of *BTN3* were found, but we were able to amplify *BTN3*-V to *BTN3*-C including a corresponding intron from genomic liver DNA using the same primers. TOPO TA cloning of this PCR product resulted in five clones of apparently two distinct types (GenBank: cl1: MG600558; cl3: MG600559; cl5: MG600560; cl4/6: MG600561). One type was strikingly like the *BTN3*-V containing contig AAGV03240336.1 and the *BTN3*-C comprising contig AAGV03240337.1, which lead us to link those two contigs together (Figure 1F). However, the three TOPO clones of this subtype were not nucleotide-identical (cl1, cl3, cl5). The two remaining TOPO clones (cl4, cl6) were identical but could not be mapped to an armadillo wgs database contig and those clones were only 92–95% identical to the previously predicted *BTN3* loci. This could indicate the existence of even more loci for *BTN3* homologs in the armadillo. Closer comparison of the two predicted *BTN3* loci in the armadillo showed an apparent deletion in the AAGV03240337.1 contig when blasted with AAGV03145787.1 (Figure 1F). The first deletion results from a fusion of a truncated exon 4 with exon 6, the second deletion includes exon 8 and the B30.2 domain encoded by exon 9. In summary, no evidence was found for the expression of a *BTN3* homolog and even in the unlikely case that expression of such a gene was missed, we do not expect that these transcripts yield functional proteins. This is especially evident compared to the loci of alpaca and dolphin *BTN3*-like genomic regions, which feature not only homologous regions to all nine *BTN3A3* exons, but are also *in silico* translatable and in the case of alpaca also expressed on cDNA level.

### *In Silico* Translatable *TRDV2* Rearrangements Are Expressed in Armadillo

**Figure 2:**
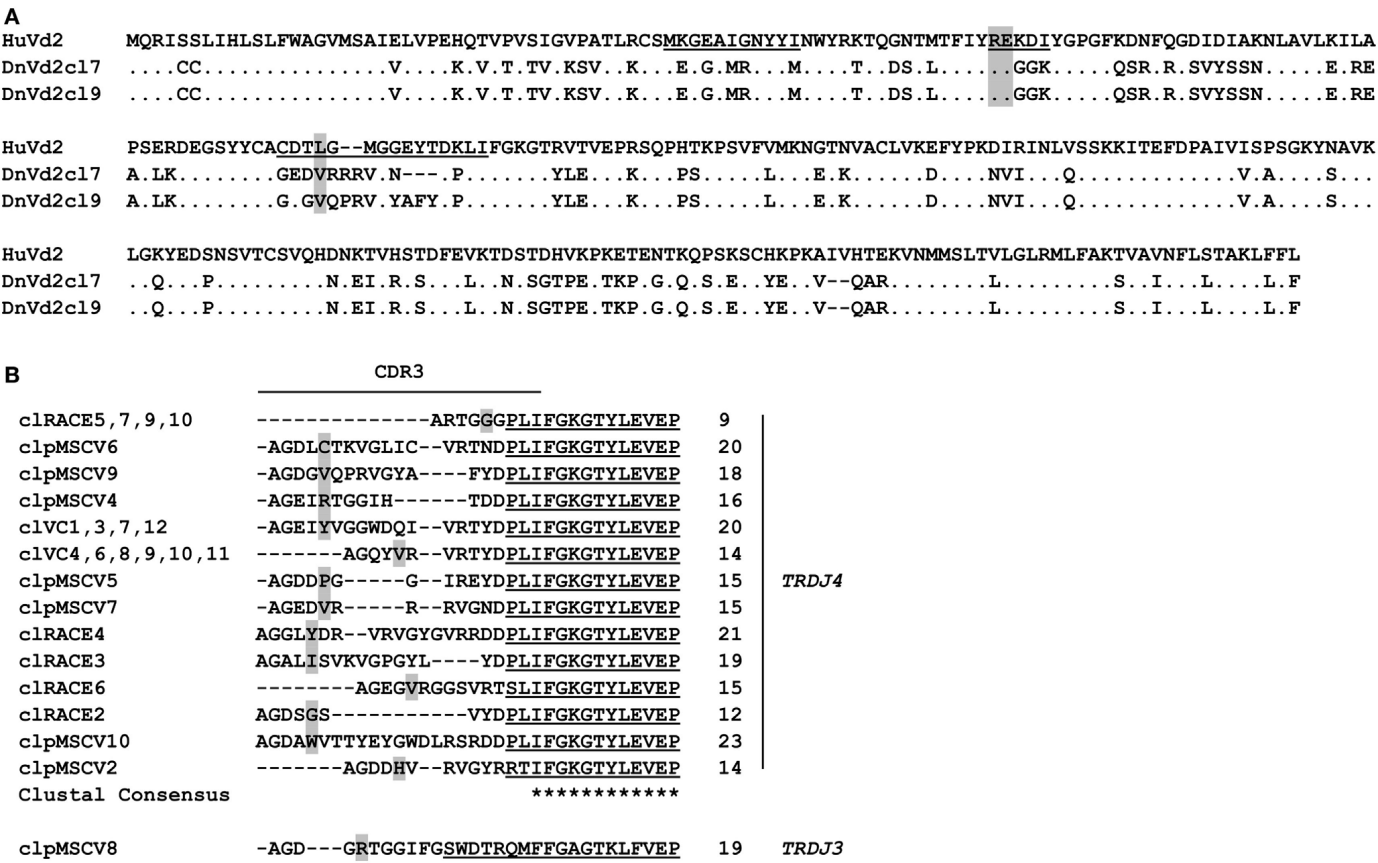
*In silico* translatable Vδ2 T cell receptor chains are expressed in *Dasypus novemcinctus* PBMCs. **(A)** Alignment of human G115 Vδ2 chain (PDB: 1HXM_A) (30) and two representative armadillo Vδ2 chains (obtained from cloning of full-length armadillo Vδ2 chains into pMSCV-IRES-mCherry FP). CDR regions appear underscored and positions δ51/52 and δ97 (gray) are highlighted. **(B)** CDR3 regions of TRDV2 clones obtained by TRDV2/TRDC PCR (clVC1, 3, 4, and 6–12), TRDV2 3′RACE PCR (cl2-7, 9, 10), and cloning (clpMSCV2, 4–10). CDR3 lengths and TRDJ-usage are indicated on the right. Alignments were calculated with Clustal Omega webtool, identical amino acids (dots), J region (underscored), and positions δ51/52 and δ97 (gray) are highlighted. The GenBank Accession numbers of unique clones are: cl2 (MG021118); clVC1 (MG021131); clVC4 (MG021132); cl3 (MG021127); cl4 (MG021128); cl5 (MG021129); cl6 (MG021130); clpMSCV2 (MG807648); clpMSCV4 (MG807649); clpMSCV5 (MG807650); clpMSCV6 (MG807651); clpMSCV7 (MG807652); clpMSCV8 (MG807653); clpMSCV9 (MG807654); clpMSCV10 (MG807655).

**Figure 3:**
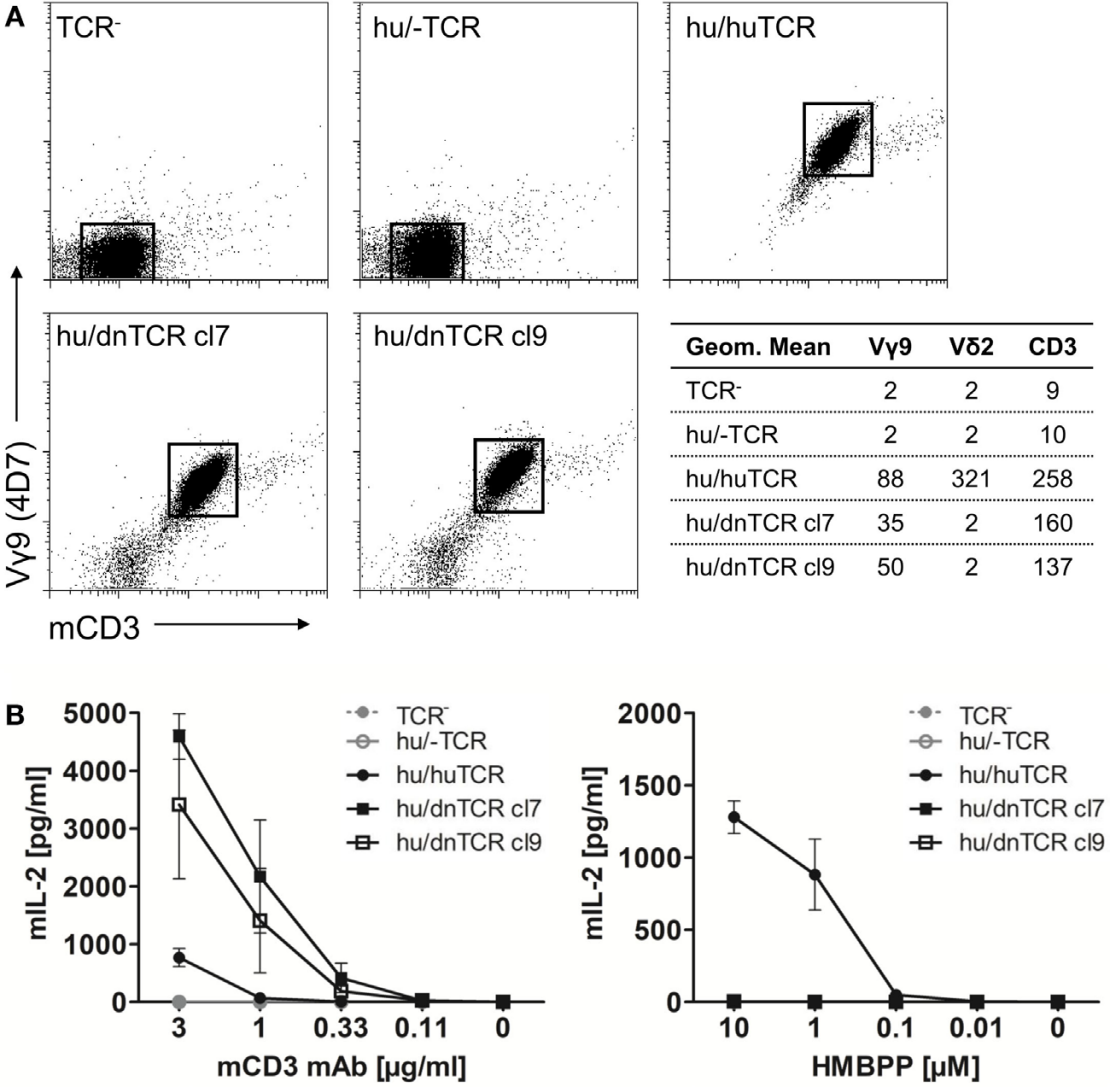
Surface expression of a functional armadillo Vδ2 T cell receptor (TCR) chain. **(A)** Armadillo Vδ2 TCR chains (pMSCV-IRES-mCherry FP armadillo Vδ2 cl7 or cl9) were retrovirally transduced into TCR-negative murine cell lines (BW58 r/mCD28). The human Vγ9 TCR MOP chain (pEGN huVγ9, GenBank: KC170727.1) was co-transduced and TCR surface expression was confirmed with flow cytometry stainings of the human Vγ9 chain, Vδ2 chain, and mouse CD3. Dotplots of Vγ9 (*Y*-axis, log) and CD3 (*X*-axis, log) co-stainings are shown and geometric means of Vγ9, Vδ2, and CD3 stainings are indicated. **(B)** BW58 r/mCD28 cells and TCR transductants were cultured for 22 h in 96-well plates coated with α-mCD3 mAb or with RAJI-RT1B^1^ cells in the presence of increasing amounts of HMBPP. Mean + SEM of three independent experiments is shown for each cell line. Stimulation of hu/huTCR with 10 µg/ml α-mCD3 mAb resulted in 651 pg/ml (SEM: 129).

In contrast to the lack of expression of a *BTN3*-like gene by *D. novemcinctus*, we demonstrate the expression of *in silico* translatable *TRDV2* TCR chains (IMGT nomenclature if not otherwise indicated). Full-length armadillo *TRDV2*-like variable regions preferentially recombined with a *TRDJ4* homolog could be assembled through the amplifications of *TRDV2/TRDC* from armadillo PBMCs, RACE PCR and cloning of full-length *TRDV2* chains into the pMSCV-IRES-mCherry FP plasmid. The overall amino acid identities of two clones carrying *TRDV2/TRDJ4* homologs to the human G115 Vδ2 chain were 65% for both clones (Figure 2A). The armadillo V region shares a 77% nucleotide and a 59% aa identity with the human G115 Vδ2 chain, the J region is 86% (nt) and 86% (aa) identical to the human *TRDJ4, TRDC* of armadillo and human show a conservation of 82% (nt) and 69% (aa). A single clone was found to carry a *TRDV2* rearrangement with a *TRDJ* region homologous to human *TRDJ3*, with 88% (nt) and 89% (aa) identity (Figure 2B). PAg-reactive Vδ2 chains in humans commonly use *TRDJ1, 2* or *3* (46), however, preferential but not exclusive rearrangement of *TRDV2* with a *TRDJ4*-like J segment has been shown in *V. pacos* (23). Other conserved features of PAg-reactive Vδ2 chains are varying CDR3 lengths (46), the residues Arg51 (30, 46, 47) and Glu52 (30), and the presence of a hydrophobic amino acid (Leu, Ile, Val) at position δ97 (46, 48). Partial armadillo *TRDV2*-like rearrangements were amplified either through 3′ RACE (8 clones) or *TRDV2/TRDC* amplification (10 clones) and PCR products were cloned with the TOPO TA cloning set for sequencing with pCR4-TOPO vector (Thermo Fisher Scientific). Another eight unique *TRDV2* clones were obtained from cloning of full-length rearranged armadillo *TRDV2* transcripts into the pMSCV-IRES-mCherry FP vector. All those partial clones were in frame with CDR3 lengths of 9-23 aa (Figure 2B). The positions Arg51 and Glu52 are conserved in all our armadillo clones and 5 out of 15 unique CDR3 sequences carry valine or isoleucine at δ97. Two armadillo Vδ2 chains amplified by PCR from cDNA were co-expressed with a human Vγ9 chain (TCR MOP) in a TCR-negative mouse cell line (BW58 r/mCD28) (33, 34). Surface expression of heterodimeric TCRs was confirmed by flow cytometry staining of the Vγ9 and Vδ2 chain and mouse CD3, as well as vector-encoded EGFP (human Vγ9) and mCherry (armadillo Vδ2) (Figure 3). CD28 expression of all cell lines was confirmed to be equal. The Vγ9 and CD3 expression of both cell lines overexpressing human/armadillo TCRs (hu/dnTCR cl7 or cl9) was significant but lower in comparison with human Vγ9Vδ2 TCR (huTCR) overexpressed in the same cell line. Thus, structural features important for pairing of armadillo Vδ2 chains with human Vγ9 chains seem to be conserved. Transduction of only the human Vγ9 chain did not result in surface expression of Vγ9 or CD3. Signal transduction of huVγ9/dnVδ2 TCRs was studied with *in vitro* stimulation assays. Crosslinking of CD3 by plate-bound anti-mouse CD3 mAb was performed as described before (23, 40) and resulted in a substantial mIL-2 production of TCR transductants but no detectable IL-2 secretion of TCR^–^ cells or cells transduced with the human Vγ9 chain only (Figure 3B). Anti-CD3 mediated stimulation of hu/huTCR reached saturation at 3 µg/ml anti-CD3 as indicated by stimulation with 10 µg/ml. Reactivity to the PAg HMBPP was not observed in a stimulation assay with RAJI-RT1B^1^ cells, although human TCR transductants (hu/huTCR) readily recognized HMBPP in this context (Figure 3B). In summary, we report functional Vδ2 chains in the armadillo, that pair with TCR γ chains and show no crossreactivity to human BTN3.

### Functional *TRG* Chain Rearrangements Lack Homologs to Human *TRGV9*

**Figure 4:**
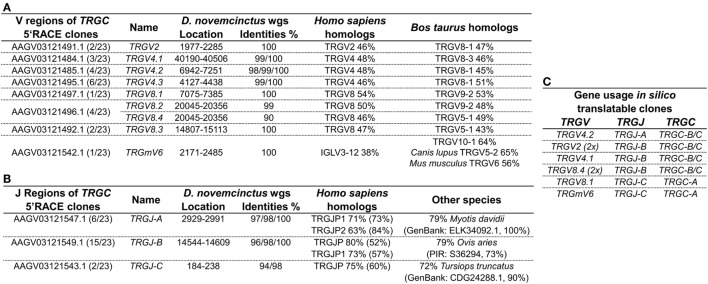
Gamma chain transcripts homologous to human IMGT subgroup TRGV1 are expressed in armadillo PBMCs. **(A)** *Dasypus novemcinctus TRGV* regions used by 5′ RACE PCR products (Accession: *TRGC-A* Ex1 AAGV03121543.1; *TRGC-B* Ex1 AAGV03121550.1; *TRGC-C* Ex1 AAGV03121548.1) with associated wgs accession numbers based on sequence homologies and location of those in the respective contigs. Armadillo *TRGV* segments were labeled according to their closest homolog in human or mouse. Varying nucleotide identities to genomic sequences and putative AA homologs in human and other species are indicated. AA homologs were determined with IMGT/DomainGapAlign (57, 58). **(B)** J regions of *TRGC* 5′ RACE clones with location in wgs contigs and identities. Designations of J regions of the armadillo are random as distinct homologs to human *TRGJ* could not be determined. Homologies were determined by NCBI blastp to AA sequence of human germline encoded J regions or the NCBI non-redundant protein sequence database for other species. **(C)** Gene usage of *in silico* translatable *TRGC* 5′ RACE clones. The GenBank accession numbers of productive rearrangements are supplied in Table S2 in Supplementary Material.

Genomic surveys revealed a *TRGV9*-like gene (Accession: AAGV03121505.1 nt402-695) in *D. novemcinctus*, which is *in silico* translatable and shares 80% of its nucleotides and 69% of its amino acids with the human G115 TCR γ. We were, however, not able to amplify a *TRGV9* transcript from armadillo PBMCs *via* PCR of *TRGV9/TRGC* or 3′RACE PCR from *TRGV9*. Notably, we found four different regions (*TRGC-A, -B, -C, -D*) homologous to the first exon of the TCR γ constant region in the armadillo wgs database. Armadillo *TRGC-A, -B*, and -C (Accession: *TRGC-A* Ex1 AAGV03121543.1; *TRGC-B* Ex1 AAGV03121550.1; *TRGC-C* Ex1 AAGV03121548.1) can be fully translated, however, *TRGC-D* (Accession: *TRGC-D* Ex1 AAGV03173223.1 nt672-373) contains stop codons and is most likely a *TRGC* pseudogene. The first exons of *TRGC-A* and *TRGC-B/C* share 94% nucleotide identity, *TRGC-B*, and *TRGC-C* are 98% identical on the nucleotide level and all of them are 80% identical to exon 1 of the human *TRGC1*. Amplification of the 3′ end and 5′RACE PCR of *TRGC*-*A/B/C* exon 1 confirmed *TRGC-A* and *TRGC-B*, but not *TRGC-C* transcripts on cDNA level. It appears that *TRGC-A* is encoded by 3 exons, which are all represented in the contig AAGV03121543.1 (exon1: nt3646-3953, exon2: nt7499–7548, exon3: nt9731-9871), whereas *TRGC-B* and *TRGC-C* are encoded by 4 exons with exon 1 and 2 in the contigs AAGV03121548.1 (nt6277–6590 and nt7441–7491) and AAGV03121550.1 (nt3170–3478 and nt7125-7178), and exon 3 and 4 in AAGV03121549.1 (nt101-152 and nt 2384–2525) and AAGV03121551.1 (nt1733-1784 and nt3978–4119), respectively. However, we were not able to assemble full-length *TRGC*-like regions from those contigs. Through 5′RACE PCR of *TRGC*, we can additionally report the existence of several armadillo *TRGV* transcripts. Of 23 clones used for the analysis (Table S2 in Supplementary Material), only eight were fully translatable, which corresponds to other findings of a multitude of non-productive TCR γ chain rearrangements, which can be expressed by cells that later commit to the αβ lineage (49, 50). The transcripts were compared to the armadillo wgs database and genomic location and accession numbers of contigs indicated the existence of nine different *TRGV* regions in our clones (Figure 4A). Those regions were found to be homologous to the human TRGV1 cluster (*TRGV1-8*) with amino acid identities ranging from 46% up to 54%. Higher similarities were found with *Bos taurus TRGV* (43–64%). One particular armadillo V segment could not be assigned to a human *TRGV*; however, it shares 56% identity with the mouse *TRGV6*. These V genes were rearranged with three different J regions (*TRGJ-A, TRGJ-B*, and *TRGJ-C*) (Figure 4B) sharing amino acid homologies of 63–80% with human *TRGJ* segments. Query cover with human homologs varied from 52 to 84%, which made a definite assignment difficult and lowers amino acid identities. Concerning the translatable clones resulting from the 5′RACE PCR (Figure 4C), it is interesting that *TRGJ-A* and *TRGJ-B* from *D. novemcinctus* seem to associate with other *TRGV* than *TRGJ-C*. Additionally, the *TRGC* usage of *TRGJ-C* is restricted to *TRGC-A*, the other J segments use either *TRGC-B* or *TRGC-C*, which could not be distinguished in this 5′RACE PCR. This apparent bias in C region usage is reminiscent of a cassette structure of the armadillo *TRG* locus comparable to artiodactyls or the bottlenose dolphin (IMGT-Locus representations) (26). Due to the lack of any evidence for a functional *TRGV9* rearrangement in armadillo PBMCs, together with the fact that we found other *TRGV* in a functional rearrangement with *TRGJ* in the armadillo, we propose the lack of expression of Vγ9Vδ2 TCRs in this species.

## Discussion

In this study, we report for the first time, an analysis of the expression of the essential components of the BTN3/Vγ9Vδ2 TCR system in the nine-banded armadillo (*D. novemcinctus*) and a comparison with homologous genes of other mammalian species. Studies of the distribution of the *TRGV9, TRDV2*, and *BTN3* genes identified this animal as a candidate for a functional Vγ9Vδ2 T cell population with a corresponding BTN3 molecule, which is essential for PAg recognition. However, we observed that aside from expression of *in silico* translatable *TRDV2* chains, the armadillo does most likely not express a functional *TRGV9* rearrangement. Surface expression of armadillo Vδ2 and human Vγ9 chains was achieved and signaling after CD3 stimulation was observed. This is an interesting finding, as apparently structural features, which allow pairing of armadillo Vδ2 with Vγ9 are conserved, although no evidence for *TRGV9* expression was found. However, pairing of Vδ2 chains is not restricted to Vγ9 TCR chains in humans (51) even though there are certain pairings of γδ chains in mice that fail to be expressed (52). PAg-reactivity of human/armadillo heterodimeric γδ TCRs could not be shown. This was not surprising given that previous alanine-scanning mutagenesis showed contribution of all six CDR3 to PAg-reactivity (46). Nevertheless, armadillo Vδ2 chains might become a valuable model for future mutagenesis and structural studies, e.g., by transplanting human CDR into the armadillo Vδ2 chain. Moreover, the fact that in a species, which lacks bona fide PAg-reactive Vγ9Vδ2 TCR a third of the clones expresses the amino acids isoleucine or valine at position 97 suggest that the common use of these amino acids might not be taken as an indicator for a certain PAg-reactivity but may be largely random or a result of selection by structural requirements or other ligands (23, 48).

In addition to the lack of evidence for *TRGV9* rearrangements, no full-length *BTN3* transcript seems to be expressed in the armadillo. Based on genomic data, we report evidence for the existence of a multigene family of *BTN3*-like genes in the armadillo. Assessment of numbers of genes and their structural analysis is not possible to this date due to lack of genomic data and transcripts. We identified one locus that closely resembles the human *BTN3A3* locus and another one carrying deletions of transmembrane domains and the B30.2 domain, which could be more like a *BTN3A2* gene. However, the lack of signal sequences and multiple deletions and frameshifts as well as the overall lack of transcripts of a BTN3-like molecule speaks against functional BTN3 molecules in armadillo. The lack of leader sequences for all identified *BTN3-V* segments might indicate that loss of function preceded the duplication events. In contrast, in primates a duplication of the *BTN3* loci occurred (20) and led to new BTN3 molecules such as BTN3A1. This isoform is not only essential for the mediation of PAg-dependent stimulation of Vγ9Vδ2 T cells but also contributes to signaling to induce type I interferon transcription (15, 53). The fact that the non-functional armadillo B30.2 domain has preserved the codons for all six amino acids contacting the PAg in the proposed PAg binding sites and the existence of a translatable, although not expressed, *TRGV9* homolog may indicate the loss of functional elements for PAg sensing by γδ T cells in the armadillo ancestor. With the armadillo as an animal model for *M. leprae* in mind (27), one could speculate that a non-functional Vγ9Vδ2 T cell subset leads to higher susceptibility for this pathogen. In armadillos, however, low core body temperatures of 33–35°C could be seen as a factor that favors *M. leprae* proliferation *in vivo* (54, 55). Furthermore, other species like rodents, which have lost the BTN3/Vγ9Vδ2 system do not exhibit higher susceptibility to leprosy manifestations (29, 56). Regarding our observations of lacking transcripts of the BTN3/Vγ9Vδ2 system, we can only state that the armadillo cannot be used as a model for this T cell subset.

So far, there are two other non-primate species that can be considered prime candidates for possessing PAg-sensing Vγ9Vδ2 T cells. First, the alpaca (*V. pacos*), which not only expresses transcripts but also possesses a Vγ9Vδ2-like cell population that expands upon HMBPP stimulation (25). This species shows not only functional rearrangements of *TRGV9* and *TRDV2* but additionally a single BTN3 molecule (23, 24). Interestingly, this more primordial BTN3 possesses the predicted PAg-binding sites of both BTN3-V and B30.2 domain of the human BTN3A1 within a protein more closely related to human BTN3A3. The second species, the bottlenose dolphin shows functional *TRDV2* rearrangements as well as *TRG* rearrangements homolog to human *TRGV9/TRGJP* containing TCR-chains and a single *BTN3*-like gene. With these candidates in mind, it seems even more likely that *D. novemcinctus* cannot be considered a model organism for PAg-reactive Vγ9Vδ2 T cells, but stands as a witness for the emergence of this system with placental mammals.

## Ethics Statement

Armadillos were maintained and samples collected in accordance with all ethical guidelines of the U.S. Public Health Service under protocols approved by the IACUC of the National Hansen’s Disease Program, assurance number A3032-1.

## Author Contributions

AF planned, performed, and analyzed experiments, and wrote the manuscript. MK reviewed the manuscript and provided the sequence for *Vicugna pacos* BTN3. LS performed experiments. RT provided samples and reviewed the manuscript. TH conceived the study, planned and analyzed experiments, and wrote the manuscript.

## Conflict of Interest Statement

The authors declare that the research was conducted in the absence of any commercial or financial relationships that could be construed as a potential conflict of interest.

## Abbreviations

BTN, butyrophilin; BTN3-V, BTN3 IgV-like region; BTN3-C, BTN3 IgC-like region; HMBPP, (E)-4-hydroxy-3-methyl-but-2-enyl pyrophosphate; PAg, phosphoantigen; wgs, whole genome shotgun contigs.
