# Supplementary material for: The Armadillo (Dasypus novemcinctus): A Witness but Not a Functional Example for the Emergence of the Butyrophilin 3/Vγ9Vδ2 System in Placental Mammals
Source: Front Immunol. 2018 Feb 23;9:265. doi: 10.3389/fimmu.2018.00265 (PMC5829056; doi:10.3389/fimmu.2018.00265)
Supplement: Supplementary file 1 [file data_sheet_1.PDF]

Supplementary Information

Supplementary Table 1: Primers (synthesized by Sigma-Aldrich) and used for the amplification of armadillo TRDV2-, TRDV9-chains and BTN3.

TRDV2/TRDC	TRGV9/TRGC	BTN3
A21 Vδ2for<br>GCCATTGTGTTGGTGCCTGAA	A17 Vγ9for<br>CTAGAGCAACCTCGACTTTCTA	A122 BTN3panIgVfor<br>TTCCAAGATGACAACTTCTATGA
A71 Vδ2for2<br>TAGTGACCGTGACTGTGGGAA	A66 Vγ9for2<br>TGTGTGGTATCTGGAGTAACAAT	A123 BTN3panIgCrev<br>AGAGCCCTCCCCAGAGCC
A29 Vδ2for3<br>TTCCGTGGCAGCGTTTATAGTT	A27 Vγ9for3<br>GGACAAATTTGAGGTGGACAAG	A163 BTN3RACE3'panIgVfor<br>CATCGTGGCCATGGTGGGTGAGGA
A23 CδEx1rev<br>AGACGACGATAGCAGGGTCAA	A19 CγEx1rev<br>TCCCATTCACGGTCAGCCAG	A164 BTN3RACE3'panIgVfor2<br>CCAGGCTCAGGCAGGTGGTGGA
A30 CδEx1rev2<br>CAACTTTCGTCTCATTTTTCATGA	A28 CγEx1rev2<br>TTGAAGGAAGAAACACAGTGGG	A165 BTN3RACE5'panIgVrev<br>CCATGGCTTTCTCATAGAAGTTGTCAT
A72 CδEx1rev3<br>TTTGTCTTCAATTCAAAGTCAGAG	A67 Cγ1-3Ex1rev<br>ACACATTTGTGTTCTTCATCCAT	A166 BTN3RACE5'panIgVrev2<br>ATAACACAGGTAGTGTCCACTGTCAGA
A73 CδEx1rev4<br>CATTATTGTGTTGAACTGAACATG	A68 Cγ1-3Ex1rev2<br>TGGGATTCCAGAATCGTATTGC	A167 BTN3RACE3'panIgCfor<br>GCGCTGGGCTCTGATCTCCACATT
A94 Vδ2RACE3'for<br>GTAGTGACCGTGACTGTGGGAAAGT	A78 Cγ4Ex1rev<br>CTCCTCTATTATCTTCATGTTTGA	A168 BTN3RACE3'panIgCfor2<br>CTGTGATGGTGAAAGGCGGCTCT
A95 Vδ2RACE3'for2<br>GAGCGATGCGTAACTACTACATGAA	A93 Vγ9RACE3'for<br>CCCGCCTGGAATGTGTGGTATCT	
A118 Vδ2RACE5'rev<br>CAGTGATGCCTCACGAATCTCGAGTA	A92 Vγ9RACE3'for2<br>CAGGTTTTCTAGAGCAACCTCGACTT	
A119 Vδ2RACE5'rev2<br>TAAACGCTGCCACGGAACCTGCTCT	A86 CγRACE5'rev<br>TGTCTGACCACACATTTGTGTTCTTC	
A193 Vδ2pMSCV for<br>CTAGGCGCCGGAATTCGCCGCCACCATGCAAAGGATCTGCTGTCTC	A87 CγRACE5'rev2<br>GGATTCCAGAATCGTATTGCCATCCT	
A194 Vδ2pMSCV rev<br>CGAGCAATTGGGATCCTTAAAAGAAAAGTAACTTGGCAGT	A103 CγRACE3'for<br>TGTTTCTTCCTTCAATTGCTGAAACACAT	
	A104 CγRACE3'for2<br>AGGATGGCAATACGATTCTGGAATCC	

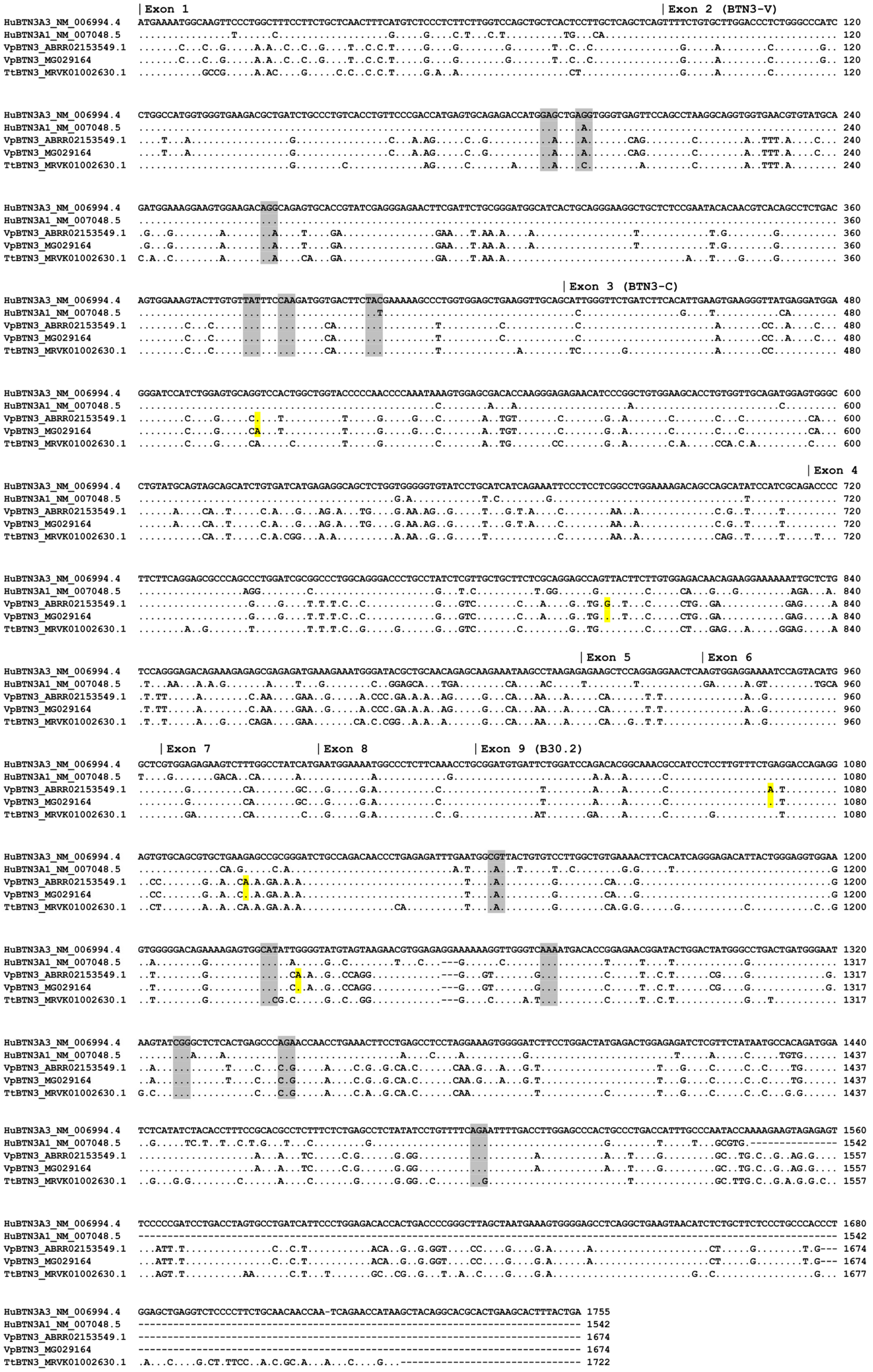

Supplementary Figure 1: Nucleotide alignment of full-length BTN3 transcripts/predicted BTN3 sequences of human, alpaca and dolphin. The BTN3 transcripts of human BTN3A3 (GenBank: NM_006994.4), human BTN3A1 (GenBank: NM_007048.5) and alpaca (GenBank: MG029164) were aligned with the *in silico* spliced BTN3-like sequences of alpaca (*Vicugna pacos* whole genome shotgun contig ABRR02153549.1) and dolphin (*Tursiops truncatus* whole genome shotgun contig MRVK01002630.1) obtained from the NCBI database. Clustal Omega was used to calculate the nucleotide alignment. Identical nucleotides (dots), gaps (dashes) and the first nucleotide of each domain (following exon organization) are indicated. Codons for amino acids implied in the recognition of phosphoantigens (Vavassori et al., 2013; Sandstrom et al., 2014) are highlighted in gray, polymorphisms found in the two alpaca BTN3 transcripts are marked in yellow. Abbreviations: transmembrane domain (TM), juxtamembrane domain (JM).

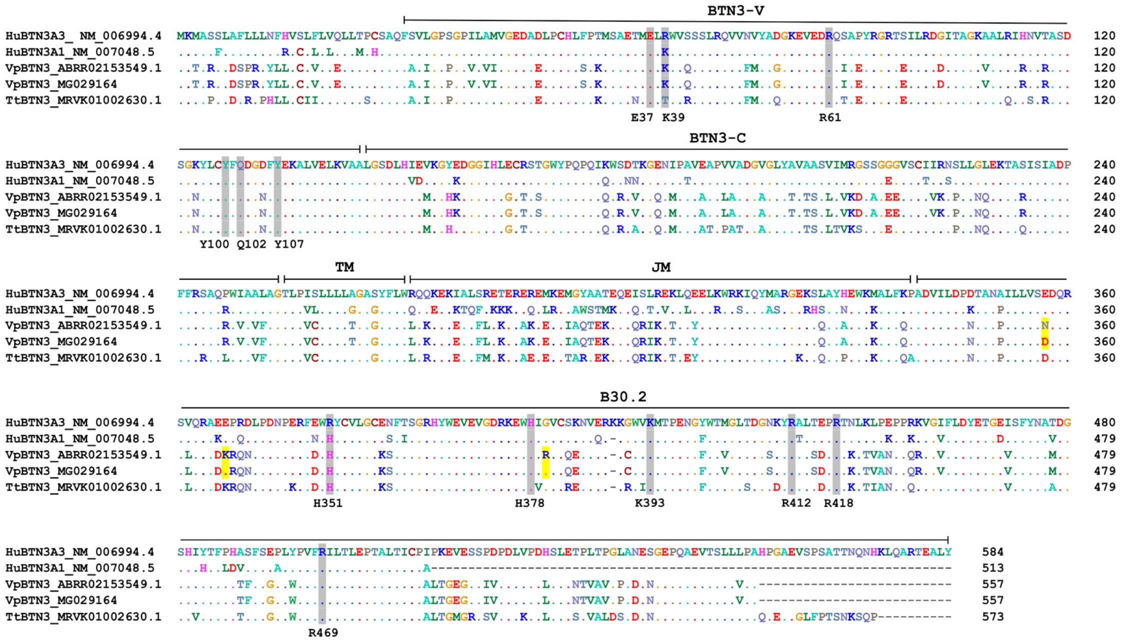

Supplementary Figure 2: Amino acid alignment of BTN3 sequences of human, alpaca and dolphin. Nucleotide sequences as described in **Fig. 1** were translated in silico and aligned using the Clustal Omega tool. Identical amino acids (dots), gaps (dashes) and polymorphisms in the alpaca sequences (yellow) are indicated. The domains of BTN3 were determined according to human BTN3A1 as described before (Nguyen et al., 2017) and proposed phosphoantigen binding residues are highlighted in gray (Vavassori et al., 2013; Sandstrom et al., 2014). Abbreviations: TM (transmembrane), JM (juxtamembrane).

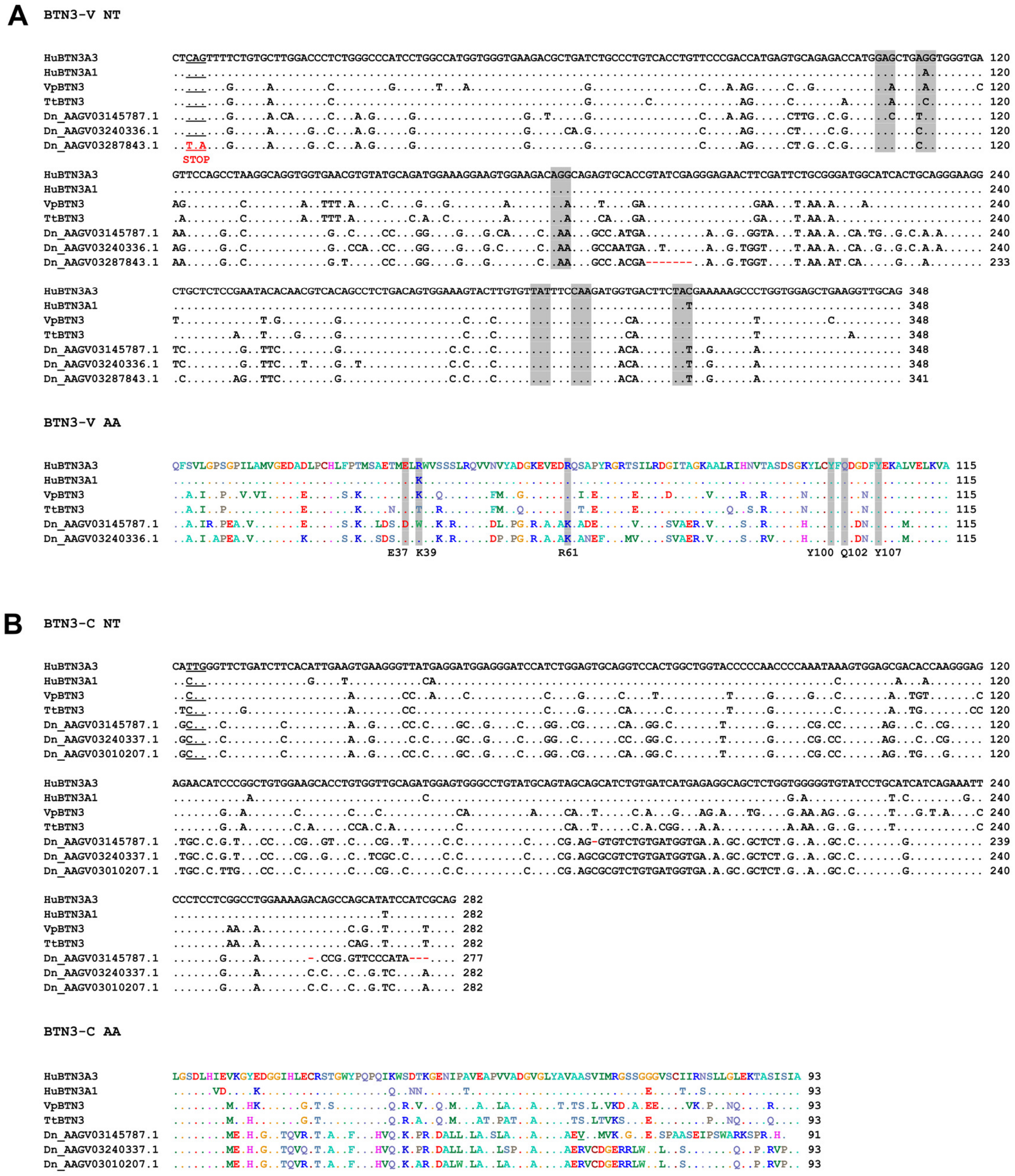

Supplementary Figure 3: Alignment of the proposed armadillo BTN3-V and BTN3-C sequences with human, alpaca and dolphin. **(A)** The BTN3-V regions of human BTN3A1 and BTN3A3, alpaca BTN3 (ABRR02153549.1) and dolphin BTN3 were determined according to the exon structure obtained via splicing prediction and aligned with three BTN3-V homologous sequences found by NCBI BLAST analysis (using human BTN3A3) of armadillo whole genome shotgun contigs (AAGV03145787.1 (corresponding to **Fig. 1E**); AAGV03240336.1 (**Fig. 1F**); AAGV03287843.1 (**Fig. 1G**)). **(B)** The BTN3-C regions were determined as described for BTN3-V and compared to the homologous armadillo sequences (AAGV03145787.1 (**Fig. 1E**); AAGV03240337.1 (**Fig. 1F**); AAGV03010207.1 (**Fig. 1G**)). The nucleotide/amino acid sequences were aligned using Clustal Omega. Identical nucleotides/amino acids appear as dots, gaps are indicated by dashes and phosphoantigen binding residues are highlighted in gray. The first codon used to *in silico* translate the nucleotide sequences appears underscored. The termination codon preventing the full-length translation of BTN3-V in the contig AAGV03287843.1 and the missing nucleotides disturbing the reading frame of the BTN3-V in AAGV03287843.1 and the BTN3-C in AAGV03145787.1 are highlighted in red.

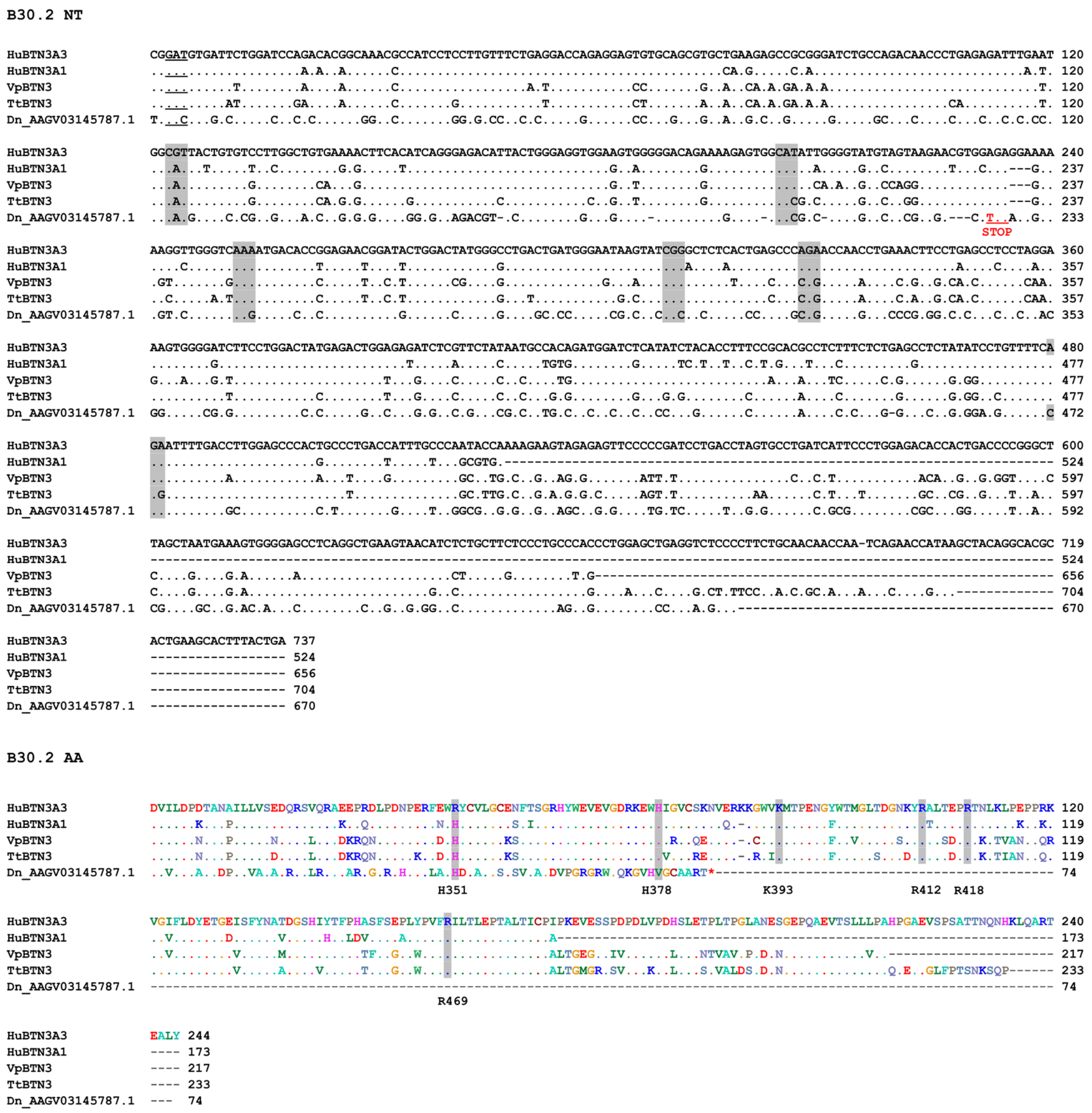

Supplementary Figure 4: Alignment of the predicted B30.2-like domain of armadillo with human, alpaca and dolphin. The single B30.2 like domain obtained from one contig in the armadillo wgs database (AAGV03145787.1, (**Fig. 1E**)) was aligned with human, alpaca and dolphin BTN3 B30.2 domains determined by splicing prediction. The nucleotide/amino acid sequences were aligned using Clustal Omega. Identical nucleotides/amino acids appear as dots, gaps are indicated by dashes and phosphoantigen binding residues are highlighted in gray. The first codon used to *in silico* translate the nucleotide sequences appears underscored. The termination codon preventing the full-length translation the armadillo B30.2 domain is highlighted in red in the nucleotide sequence and indicated in the amino acid sequence (*).

Supplementary Figure 5: Organization of genomic regions in the *Dasypus novemcinctus* contigs (**A**) AAGV03145787.1 (**Fig. 1E**) and (**B**) AAGV03240337.1 (**Fig. 1F**). The human BTN3A3 nucleotide sequence was used for a NCBI Blast search in the armadillo wgs database. The exons 2, 3, 4, 6 and 9 (highlighted in color) could be determined by this in the contig AAGV03145787.1 and the exons 3 and 4 (highlighted in color) in the contig AAGV03240337.1. The remaining exons were found by NCBI Blast of human non-coding regions between exon 4 and 9 to the armadillo contig AAGV03145787.1. The exons 6 and 7 in AAGV03240337.1 were assigned via homologies to AAGV03145787.1. Partial alignments for this are shown below. The exon-intron organization (marked by //) was determined by consensus splice donor and acceptor sites or homologies to human BTN3 exons. No termination codon was identified for exon 9 in contig AAGV03145787.1 and no splice site could be determined for exon 4 in the contig AAGV03240337.1.

A *Dasypus novemcinctus* Contig AAGV03145787.1

Genomic regions homologous to:
Exon 1 (not found), 2 (BTN3-V), 3 (BTN3-C), 4, 5 (not found), 6, 7, 8, 9 (B30.2)

>AAGV03145787.1 Dasypus novemcinctus isolate 3-136 Contig145889, whole genome shotgun sequence (length: 29470; region shown: 1855-8962)

```
ATTGGGCCATGTCATTAGGGCATTTTGTCCCCTGGCAGCCCCAGCTCCAGCCCCCTCTAATGGCACGTCCCCCTTTTGCTGAGCAG//CTCAGTTT
GCTGTGATCAGACCCCCTGAGGCCATCGTGGCCATGGTGGGTGAGGATGCTGAGCTGCCCTGTCACCTGTCCCCAAAGATGAGCTTGGACAGCATG
GACCTGTGGTGGGTGAAATCCAGGCTCAGGCAGGTGGTGGACCTGTACCCAGGGGGAAGGGAGGCAGAAGCCAAACAGGCCGATGAGTATCGAGGA
AGGACGGTAATTTTAAGAGACAGTGTCGCCGAAAGGAAGGTCGCTCTCCGGATTTCCAACGTCAGAGCCTCTGACAGTGGACACTACCTGTGTTAT
TTCCAAGATGACAACTTCTATGAGAAAGCCATGGTGGAGCTGAAGGTTGCAG//GCGAGCCCCTGGCTTTTGCATAAGCTCCTTCCCCTGTGAGAC
CTGGGGTGGGTACAGGCTTCGGGCTCTCAACCCACCTGCAGCCCTCCTGGTAGCCACGAGGAGCTGCCACCATTGCCTCCCGGTAGCCACAAGGAG
CTGCCACCGTTGCCTCCTGGTAGCCACGAGGAGCTGCCACCATTGCCTCCTGGTAGCCACGAGGAGCTGCCACCGTTGCCTCCTGGTAGCCACGAG
GAGCTGCCACCGTTGCTTCCTGGCAGCCACGAGGAGCTGCTGCCGTTGCCTCCTGGTAGCCACGAGGAGCTGCCGCCGTTGCCTCCTGGCAGCCAC
GAGGAGCTGCCGCCGTTGCCTCCTGGTAGCCACGAGGAGCTGCCGCCATTGCCTCCTGGTAGCCACGAGGAGCTGCCACCGTTGCCTCCCTGGGCT
TAAGAAGGAGACCTGGGCTTTCTACCAGGAAGAAGTCCTACTGCAGCCGACATGGTCAAGCTCAGAACTCCATCATGGTGGCCAGCAGGGGTCCAA
GCAGGGAAGGAGAACGAGAGGTGGTTGGTGGGGTGTTCTGGTTCAGGGGTGGGCTGGGGGCCTGTATGACTGTTCTCCAGCCAGGGGTGTCACTGA
GGGAGAAGGCCCACCATGGGAGGCCGGGGGCCTCAGGAAGAGGGTGTCAGCCTGGTGTTAGGGCCTTTGGGGGGTGGTTCACACAAGTGGGGCCGC
TGTCAGGAAGGAGGGGGTAATTCTGGGAGTGGGTCTCTTAGTAAATCTCACCTAGGGGGAAGGAAGACGAGGGCGAGGGTGAGGCTGAAGCTGTGA
TGGGTGGTGAGGCTGCACGGTCATCTTGTGGTGGCTCGTGGTTGCCTCCACGCTCAGCCACCGCTGGGCAGTGCTCTTGTTCCCGTGATAATCTGT
AAAGGTGCCAAAGCGCACCATTGGACATGATGCTCGGAGACATTGTGTCTGTTCAACAGCGCTGTGAGTGCCAGGCCGGCTCCCGGGTGCCAGGGC
TGCCTTTCTCCGTTCTCAGGCATGCCCTTGCCCTGCGGCTTCTGGTGGGGTTTGGTCCATGGGGAGCCCCAGCAGGAGGTGGAGGGAGGGAGAAAG
GAGGCAGCGGTGCCTGGAGCTGGCAGCGTCCTCTCCAGGACTCTCCTCTTGGGGCTTCGGAAGAGCGTCCTCTCCTCCTCTCTTGGAGCTTTGGGG
TGGCCGCTCTGCTCTCGTTGGCCCTGGGGTGCTGCAGTACCCCATGCCATTTCCCCTCACCACTCCCACTTTGGGAATCCGCTCCTTAGTTCAGTC
GCTCTGAATGAGTTCCTGTGAGTGAGCCATCGGGTCTCCTTGCGGCCCTGCCTGGTGCCGAAGGCTCCCAAGAATTCAGCACGTGCTTCCTCCGCA
G//CGCTGGGCTCTGATCTCCACATTGAAATGGAGGGCCACGAGGGCGGGGGGACCCAGGTGCGGTGCACATCGGCCGGCTGGTTCCCCCAGCCCC
ACGTCCAGTGGAAGGACCCCCGGGAGATGCCCTGCTGGCCCTGGCGGCGTCTCTGGCGGCTGATGGCGCGGGCCTGTACGCAGCGGAGGTGTCTG
TGATGGTGAAAGGCGGCTCTGGGGAGGGCTCTCCTGCAGCATCAGAAATTCCCTCCTGGGCCAGGAAAAGCCCGCGTTCCCATAGCAG//GGCAGT
GCCCGCTCAGCCTCGGCGTGCTCTGCCAGCTGGGGCTGCTGACAGGAGGAGGTGAGGCTGAGTCCCAGCTGCTGGTTTGGGCCTTTCCAGGACCTG
GAGGCTTCTCCTTGCTCCAGGGACGCTTCTCTCCCCCATAAAACCGTTGTGCCACAGGCGTTTCCTCTGTCCTGTGTACTGGAAACTGTGGCAGGC
AATGGGAATTGAACTGAAATAGTTAAGATCAAATCTTTAAACATGTCAAAGGATGGAATGCAAAAAAATAATTACAACTCCTATTACAAGGAAAGA
GCTAAGCCACTTAGGATAACTCAGTAGAAAAATGAGCAAAGAGCACGAATCATTAATTCTCAGAAGCAGAAATATACTCAATCTCATGCTTTGGAG
AAATGTATATTAAAACTTTTCCATCATGAGGTTGGCAAAATTAAAAGGGGGAAGAGGTTGATAACATATTCTGATGGCAGAGAACATGGGGACGCA
GGCCCTCTTACGCATTTGTTGATGTAATTGGTGTATTTTTGGTATAAATTGGTACAGCCTCTATGGAGGACAATACAGTGATTATTCAAACTACTT
GTTCATTGATCTGAAATCCTGTTTCTGGGCATTTGTCTGGCTGATACGTGGGCACTTGCATGAAATGAGTCATTAAATGGCGATTTATTATACTAT
TGAGGATAAGAGTGAAAAGTTAGAAACGGTGGAAACGTTCCTCAAGAGGGGCTTCTAGTGTGGAATATGACACAGCAGTGAGGAAGGACACACGGT
CCCTAACACGGAAAGTTCCCTGAGCTGTACCATGAACAGGAGAAGCAAGATTCAGGCCAGAGCGTGTAGAAGCCTGTTTTGGTGTTAAAATAAGAA
TATAGAATGGTTATTTATAATTTTTTAAACGTAACAAACCCTTGGATAGCACTCACTGTGTGCTGGGCACTTTATAAACAGTGATTTGTTGTTGAT
TACATTTTAGTTTGTTTTTTTTTTAAAGAGGCTTTAGGTTACATAAATGTTTAATTCCCTCTGACTCCGTGTAGTAGGTGCCATTACCACTCCCAT
TTTACAGAGAAGGACACTGAAAAACAGAGAGGCTAAGAAACTAGTCTAGGGTCACACTTCCACCGCAAGGAGCCAATTTAAACCAGAACTTAAACC
AGGGCCCTTCTGGCTCCGGAGTTCCCCAAGCTGACTAATAAGCTACTAAGTGGGGGCGGAAAGGCTGGGTGGATGGAGGAATTGGACGATTTGGGG
TATATTTCTGTGATTTGAACCATGTAAATAAGTTCAGTATTTGAAAACTTAAATTATGTTTTAAGTTTAAGAAACTTAAAATATTTAAGAAAACTG
TCTGATTGATGCTCGAGGAGCTCTTTCCACGGGGGTGGCAGCACTTCTGGCTGGGCCGCAGGTTGTACCAGTTTCCCAGGCGCCTGGTCCCCAGTG
CTCCCAGACCTCAGGAAAAGGCAGCGCAGTTCACCTGTGGATAACCACGGGCTCCCTGCCCTGCGGGAAGAGGGCCAAGGCCATGCGGCCCCCCAC
CCTGTCCCTCTTGCAG//ACCCCTTCTTCCAGAGCGCCGGGCCCTGGGTGGCAGCCTTCGCGGCCACGCTGCCCACCCTGCTGCAGCTCTCGTGGG
AGCCGGTTACTTCCTGTGACGACAGCAGAAG//GTCCAAGAGGCCGTAGTCCAGGAGAAAGAGAGGGCGTGTGAGAGGAGGAGGAAAAGGAACGGA
CAGAGAAGGAGAGGGAGGGAGTGGCGTGCGCATTTAAGCGCCTTCATGACTCCTCGCCCTTTATTCCTTGCAG//AGTGGAGGAAGGTCCAGTACA
TGACTC//GTGAGTGGCGCCGGCATCTCCTGAGTCAGGACTCTCCTCTCTGTCACTTTGCAGGCCCTTTCTCTTTAGCCAGGGCTGGGAAGGGCCC
TGGAGCCCTTTCTGGGGGAAGAAAGTTAGGATGGCTGATTGCAGGAAGGACAGGGGGAGCTTTTCTTTCTATAAAGGAAAAAAAACTACCGTCCA
TCTCTCTCCACAAGAGGCCCACATGCTCTCTAGCCCTGCGAGATGCTGAGGAATATCTAGGGGTGCACTTCAAATAAAGGGGCTGAGGAGGATGGA
GCCCCGTAGGTGAGCTGGCCTCCTTGTGCCAGCAGACTGCCAGCTGCTTTACATCTCTGGAGGCTGCAGAGAACTTAAAAAAAGGAAAGATTAAAA
GGACTGAGGAAGGCAGAGACGGAGGATGGGGAACAGTGTTTCTCATGAAGAGAAGAGTGGCGCAGTGGTTACAGATTTATTGCACCCGCCTTTAAA
AATTAGTGTCTGCAATGGCCTCATCAAGTTTCTCAAATTCTTTTCTGAAATAAGCGGAAACAATATAAATTGAGAAAGATGGATTCTAGTATACC
TACTGTGTGCAAGCAACACCTGCACCCAGGTATGGGGCACACACGGTGCCCTACTCGGTGCCTGTGACAACATCAGCTCGTGAGCGGTGCTCGCCA
GCCTGCTGTCGGGGAGGAGAGGGGATGGACTTGAACCTGCAGAGGCAGGAGGTCGGGGTGGGCTGGGCCTAAGGAGAGGCTGGAGGGAGGCGAGGG
GAAGAGAAAGGCAGGGAGTCAGCACACGTGGTCCAGGTGACAGTTCCGCCTTTCTCCCAACGCAG//GAGGGAAGAAGTCCCCGCACTACCATG//
```

- continued on next page

- continued from page 6

GTGAGTGGTACTGACTTTGCAGGGGCACTGAGCCCTGACACGTTCCTCCGTGGTCAGGGCTCCCAAGAGAAATAATCACATCCAGCACAGAAGCTC
CACGCAGGCACCCAGCCGTGTCTGATCTGTCAGCGCTCTGCAGAGGGGAGGCATTGGGAGATATTCCGAAGGGAGAGAGAAAAGAAAGAAAGAGTG
TTTTCCGTGGGAGGGGTGACACCTGCTTTTCGAGACCCGAGAGGAACTTCCAGATGCAGCCTCCGTGTGTCTTTGGCTGTAGCCGCGGCTAGCGCA
GGAGAGGTGGGGGGTGAGCCTAAATAATGGCCTGTTTTTCATCTCGCCTCCTTTTCAG//AGTGGAAGGCGGCCCTCTTCCAAGCTG//GGGAGTG
AAGTGCAGGCTAATGACCAAGGGACCTTCCCCCTTTTCTCCTCCAACTCCTGAGATCCTGGGGAAAGAAGCCATTGCACCCTGCGCCTTCCAGCTG
AGAGCCCAGAGAGTCAGGGAGAAGGGCTCTCCTCTGTGGGTGGAGCCGGGGAAAGGGAGGGAGACAGAACTGGAATGCAGGGAGAGCGAGGTGACC
ATGACGCAGGAGAAGGCAGTGTGCCAAGAGCGCAGCCTTCCCATGGCCCCCCACTAACTCCTCGAGTTACCTAAGGCCTCTGTGCTTTAGAATCCT
CATTCTTAGGTAGTGCGCATAATTACCAGTTCACAGGGGTTGGGGTGAAGACTGAATGAGGTCAAGGCTGAAGGGGAGGGGAAGGGCCATTCGGGA
GCCAAGGGGGCCCTGGTCCCAGGGGAGGCCTGCGGGCAGCAGGCTTGCCTTGCTTAGCTGCTGGCAGGGAGGAAGCAGGGAGAAGAAGGGCTCTGC
TCCTCTCTCTTTCCTGTTCCAATCTTCTGCCAAACCCAACAGGAAAGCAGAGGCAAGGGAGCTGTCCAGGAAGTCCTATTCCTAGAGCTAGGATGA
GGGTGGAAAAGGGTGGAGAGGGCAGTGGAGCACAAACGCCCCACCTGGTCCCGAGTACAGAGTAGGCATGGCAGGGCTGCAGCCTCCGGCACGTCC
TCGGGTGTGCCCTTCTCTAGTGTGTTCCCTGTGTTAGCACAGTCAACCCCAGAGAACGGTCTTAGCCCCAGTTTTCAGATGAGGAACAGGAGGCTA
GAGGTACGTGGGTGTCATCTGAGTTGGATCTATGGAAAGAGCATGCATTGTTAAAAGGAAAAGCCTCCCAGCCAGGCGTGGCAGAGCTGAGGCTAA
AAGCACAGGTGGCCAGAGCCAGTTGAGGAACAGGAAGCAAGAAAGTGGAAGAAGGGCGCCGTTCTGCCACGTGAGCCCACGCCAGGTCAGGAAGGC
GTGTGTGGCATCTCACCACCACCCCCTTCACACGAACACTGGACCTCCCAGCTTTCTCTCTGGGGGTTGCAGGGAGGATCACTTTCAGGGCAGGCT
GGGGACCCCAGGCTCTGGACTCGGGCCCTGGAGGCTCTGAGGTCCAGGCAGACGGGCTGAGCCCACGGGCCACCCTCGAGCCCCTCCAG//TGGAC
GTGGTCCTGGACCCCGCCACGGCGGACCCCATCCTGGTGGCCTCCGCGGACCGGAGGAGCCTGCGGCGGGCAGAGGCGCGGCGGGGTCTGCGCGAC
CACCCCGAGCGACTCGCCTGGCATGACTGCGCGCTGGGCAGCGAGAGCGTCACGGCGGGAGACGTTCCTGGGAGGGGGAGGTGGGGACAGAAAGGA
GTGCACGTCGGGTGTGCAGCGAGGACGTGAAGAGAAAGTGCTGGGTCAAGATGACCCCCGAGAACGGATGCTGGACCATGGGGCTGAGCGCCGGGA
ACGACTACCGCGCCCTCACCCAGCCGCGGACCAAGCTGCCCGTGGCCGCGCCCCCCAGACGGGTGGGCGTGTTCCTGGACCACGAGACGGGCGAGG
TGTCCTGCTACGACGCTGCCGACGGCTCCCACCTCTGCACCTTCCCGCACACCTCCTCCTCGGGCCCCTGTGGACGGTTTTCCGAATTTTGACGCT
GGAGCCCACCGTCCTGACCGTTTGTCCGGCGCCGAGAGGAGAGCAGGGGTCCCTGGTCCCTGATCTGGGGCCTGACCGCGCCCTGGAGCGCCCAGG
GACCCTGGACTCGGCTAGCGAGAACGAGGACCCTCAGGCCGAGGTGAGGTCCCTGCTTCTCCCTGAGCAGCCTGGAGCCCAGGACGCCCGCCCCGC
CCACCTTAAGTCGCTGTGGGCTCGCGGGTTCCAAGCTCCTGCTCCCAGTACTTCAGCTACGCCACGTAACGACTCTGTGACTCTGGTACCCCTTTA
GTACCCTCTTTTTTTATTTTTAATGAGTAT

Identification of homologous exons by comparison of genomic human BTN3A3 with armadillo contig AAGV03145787.1:

Exon 7: NCBI BLAST of human and armadillo contig AAGV03145787.1 non-coding region upstream of B30.2 and downstream of exon 6

```
AAGV03145787.1    AGGAGGGAAGAAGTCCCCGCACTACCATGGTGAGTG
                  ||| ||  |||||||   |  ||| |||||||||||
HuBTN3A3          AGGTGGAGAGAAGTCTTTGGCCTATCATGGTGAGTG
```

Exon 8: NCBI BLAST of human and armadillo contig AAGV03145787.1 non-coding region upstream of B30.2 and downstream of exon 6

```
HuBTN3A3          AAATACTGACCTTTTTCTTATCTGTGTCTCCTTCCTTTCAGAATGGAAAATGGCCCTCTT  399
                  ||||| || ||| ||| | ||||  | ||||||   |||||| |||||   |||||||||
AAGV03145787.1    AAATAATGGCCTGTTTTTCATCTC-GCCTCCTT---TTCAGAGTGGAAGGCGGCCCTCTT  349

HuBTN3A3          CAAACCTGGTGAGTAAATCACTGTATGTTCCCTGGATCAACAACCTGAGGGACTATATTC  459
                  | || |||| |||| ||        ||    | || | ||  |||  ||||||  |   |
AAGV03145787.1    CCAAGCTGGGGAGTGAAG-------TG----CAGGCT-AATGACCA-AGGGACCTTCCCC  396

HuBTN3A3          CTTTCTCCTCCTCCAACTCTTGTGAT  485
                  ||||   |||||||||||| || |||
AAGV03145787.1    CTTTT--CTCCTCCAACTCCTGAGAT  420
```

B *Dasypus novemcinctus* Contig AAGV03240337.1 (**Fig. 1F**)

Genomic regions homologous to:
Exon 1 (not found), 2 (BTN3-V, corresponding exon 2 in AAGV03240336.1), 3 (BTN3-C), 4 (truncated, deletion), 5 (not found, deletion), 6, 7, 8 (not found, deletion), 9 (B30.2, not found, deletion)

>AAGV03240337.1 Dasypus novemcinctus isolate 3-136 Contig240580, whole genome shotgun sequence (length: 24370; region shown: 716- 3834)

```
GGGAAGGGCTCCTTAGTTCAGTCGCTCTGAATGAGTTCCTGTGAGTGAGCCATCGGGTCTCCTTGCGGCCCTGCCTGGTGCCGAAGGCTCCCAAGA
ATTCAGCACGTGCTTCCTCCGCAG//CGCTGGGCTCTGATCTCCACATTGAAATGGAGGGTCACGAGGGCGGGGGACCCAGGTGCAGTGCACCTC
GGCCGGCTGGTTCCCCCAGCCCCACGTCCAGTGGAAGGACCCCCGGGGAGATGCCCTGCTGGCCCTGGCGGCGCCTCTGTCGCCCGATGGCGCGGG
CCTGTACGCAGCGGAGCGCGTCTGTGATGGTGAAAGGCGGCTCTGGGGAGGGCTCTCCTGCAGCATCAGAAATTCCCTCCTGGGCCAGGAAAAGCC
CGCCCGCGTTCCCATAGCAG//GGCAGTGCCCGCTCAGCCTCGGCGTGCTCTGCCAGCTGGGGCTGCTGTGAGGAGGAGGTGAGGCTGAGTCCCAG
CTGCTGGTTTGGGCCTTTCCAGGACCTGAGGCTTCTCCTTGCTCCAGGGACGCTTCTCTCCCCCATAAAACCGTTGTGCCACAGGCGTTTCCTCTG
TCCTGTGTACTGGAAACTGCGGCAGGCAATGGGAATTGAGCTGAAATAGTTAAGATCAGATCTTTAAACATGTCAAAGGACGGAATGCAAAAAAAT
AATTACAACTCCTATCACAACAAAGAAGGAAACCTCCTTAAAACAGAAAAGCTCTTACGACAACTCAGAAGAAAAATGAGCAAAGAACATGAATTA
TTCACTCTCAAACAGAAATACACTCAATCTCATGCTTTGGAGAAATGTATATTAAAACATTTTCCATCCTGAGATTGGCAAAATATGAAATTAAAA
GGGGGAAGAGGTTGATAACATATTCTGATGGCAGAGAACATGGGGACGCAGGCCCTCTTACGCATTTGTTGATGTAATTTGGCATATTTTTGGTAT
AAATTGGTACAGCCTCCATGGAGGACAATACAGTGATTATTCAAACTACTTGTTTCTTGATCTAAAGTCCTATTTCTGGGCATTTGTCTGACTAAT
ACATGGGCACTTGCATGAAATGAGCCATTAAATGACTATTTATTATACTATTGAGGATAAGAGTGAAAAGTTAGAAACGGTGGAAATGTTCCTCAA
GAGAGGCTTCCAGCATGGAATACGACACAGCAGTGAAGAAGGACACACAGTCCCACCACGGAGAGAGCCCTGAGCTGTGCCATGAACAGGAGAAGC
AAGATTCAGGCCAGAGCATTTAGAAGCCTGTTTTGGTGTAAAAATAAGAATATAGATTGGTATTATAATTTTTATATGTAACGAACCCTTGGATGG
CACTCACTGTGTGCCAGGCACTTTACAAACAGTGATTTGTTGTTGTTGTAGATTACATTTTACTTTTTTTCTTTAAAGAGGCTTTAGGTATCATAA
ATGTTTAATCCCTCTGACTCCCTGAGATAGGTGTTATTACCACTCCCATTTTACAGATGAGTATGCCTGCGGAACAGAGAGGCTAAGTAACTAGTC
TAGAGTCACACATCCTCAGCCAGGAGCCAGAATTTAAATCGTTGCCCTCCTGGCTCCAGAGTTCCCCACACCAACTAATAACAAGCTACTAAGTGG
GGAGGGGAAAGGCTGGGTAGATGGAGGAATTGGACGACTTTGGCTATTTTTCTGTGATTTGAACCATGTAAATGAGTTCAGTATTTGAAAAGTTAA
ATCAGAAAACTGTCTGATGCTCGAGGAGCTCATTCCATGGGGGCCGCAGGTTGTGCCAGTTTCCCGGGCGCCTGGTCCCCAGCGCTCCCAGACCTC
AGGAAAAGGCGGCCCAGGTCACCTGTGGATAACCGTGGACTCCCGGCCCGGAGGGAGGAGGGGCGAGGCTTCGAGGACCCCCGCCCTTTCCCCCTG
GCAG//ACCCCTTCTTCCGGAGCACCGAGCCCTGGGCGGCGGCCTTCGCGGCCACGCTGCCCACCCTGCTGCAGCTCCTCGCCATTTATTCCTCGC
AG//ATGGAGGAAGATGCAGTACATGACTC//GTGAGTGGCGCCGGCACCTCCTCTGAGTCAGGACTCTCCTCTTGATCACTTTGCAGTCCTGGAG
CCCTTTCCCCTGGGGAAGAAAGCTAGGATGGCGATTGCGGGAAGGAGAGGGGGAGCCTTTCTTTCTTTAAAAAGAAAAGTGCCGTCCATCTCTCTC
CACAAGAGGCCCACAGGCTCTCTAGCCCTGCAAGATGCTGAGGAATATCCAGGGCTGCACTTCAAATAAAGGGGGTGAGGAGGATGGAGCCCCGTA
AGTGAGCTGGCCTCCTTGTGCCAGGAGCCTAGTGAGCTGCTTTACATCTCTGTAGGCTGCAGAGAATTTAAAATAAGGAAAGATTAAAAGGAATGA
GGAAAGCAGAGACAGAGGATGAGATACGGTGTTTCTCATAAAGAGGAGAAGAGTGGTGCAGTGGTTACAGATTGCACCTGCCTTTAAAATTAGTGT
CTGAAATGGCCTCGTTGAGTTTCTCAAATTCTTTTCCAAAATAAGCGGGAAACAATATAAATTGAGAAAGACGGATTCTAGTATACCTACTATGTA
CAAGCAACACCTGCGCCCAGGTATGGGGCACACACAGTGCCCTACTCGGGTACCCTGTGACAACATCAGCTCGTGAGCGGAGCTCCCAGGCCTGCT
GTCGGGGAGGAGAGGAGATGGACTTGGACCTGCAGAGGCAGGAGGTCGGGGTGGGCTGGGCCTAAGGAGAGGCTGGAGGGAGCAAGGGGAAGAGAA
AGGCAGGGAGTCGGCGCGCGTGGTCCAGGTGACAGCTCTGCCTTTCTCCCAACGCAG//GAGGGAAGAAGTCCCTGCACTACGCTG//CTGAGTGG
TGCTGACTCCGCAGGGGCGCTGAGCCCTGACACTTTCCTCCTCGGTCAGGGCTCCCAAAGGAATAATCACATCCAGTACAGAAGCGCTTTTCGGAG
GGAGGGGCGATGACTGCTTTTTGAAACCCAAGAGGGACTTCCAGATATGGCCTCTGGGTATTTTGGCCACAGCAGCTCCTGGTGCACAGGCACGCA
CCCAGCTGTGTCTCATCTGTCAGCATTCTGGAGTGGGGAGGCACTGGGAGATATTCTGAA
```

<u>Identification of homologous exons by comparison of contigs AAGV03145787.1 and AAGV03240337.1:</u>

Exon 6: NCBI BLAST of armadillo contigs AAGV03145787.1 and AAGV03240337.1

```
AAGV03145787.1          TTTATTCCTTGCAGAGTGGAGGAAGGTCCAGTACATGACTCGTGAGTGGCGCCGG
                        ||||||||| ||||| ||||||||| | |||||||||||||||||||||||||||
AAGV03240337.1          TTTATTCCTCGCAGA-TGGAGGAAGATGCAGTACATGACTCGTGAGTGGCGCCGG
```

Exon 7: NCBI BLAST of armadillo contigs AAGV03145787.1 and AAGV03240337.1

```
AAGV03145787.1          GCAGGGAGTCAGCACACGTGGTCCAGGTGACAGTTCCGCCTTTCTCCCAACGCAGGAGGG
                        |||||||||| || | ||||||||||||||||| || |||||||||||||||||||||||
AAGV03240337.1          GCAGGGAGTCGGCGCGCGTGGTCCAGGTGACAGCTCTGCCTTTCTCCCAACGCAGGAGGG

AAGV03145787.1          AAGAAGTCCCCGCACTACCATGGTGAGTGGTACTGACTTTGCAGGGGCACTGAGCCCTGA
                        |||||||||| |||||||  || |||||||| ||||||  |||||||| |||||||||||
AAGV03240337.1          AAGAAGTCCCTGCACTACGCTGCTGAGTGGTGCTGACTCCGCAGGGGCGCTGAGCCCTGA
```

Supplementary Table 2: List of clones of the TRGC 5‘RACE PCR product. Clones were obtained through pCR4 TOPO TA cloning of the TRGC 5’RACE nested PCR product. The leader sequence is highlighted in gray and the J segment appears underscored. Accession numbers of *Dasypus novemcinctus* (D.n.) wgs hits (NCBI Blast) are indicated together with nucleotide identities

TRGC 5‘ RACE clone (GenBank Accession number, productive rearrangements only)	*D.n.* wgs Hits (Identities)	Name	Translation	Notes
>2<br>ATGCTGTGGGCTCCAGCCCTCCTTTTAGCTTTCCTGGCTCCTGCCAGTCAGACAGCTTCCAACTTGGAATGGAG<br>TGGGGAAGACTACCACCAAGCGGACTGGGGAAATGGCCACGATCACTTGTGATATAGCGAAAGAAAGCTTTAAC<br>TATCTCCACTGGCACCTGCTCCAAGAGCGTGAGGCCCCCAGACGTATTCTCTACTACGCTCCGTCTTCCGGAAA<br>GTTTTGGATAGACTCGGGAATCAAAGAAGGGAAATATCGTGCTTATAAAGGTTCAGGGAGGAGCTACAGATTCG<br>AGGTCAGGAATCCTGAGGAAAGTGATTCCGGTGTGTATAACTGTGCAGCCTGGGAGCTGCTAGCTCAGGAGTGA<br>TCAAGATTTTTGGACAAGGGACTAAGCTCACAGTAATTCCTTCTGGTGGACACCGATCCCCAAAACCCACT	V: AAGV03121495.1 (L 100%, V 99%)<br>J: AAGV03121549.1 (100%)<br>C: NA	V4.3<br>JB	MLWAPALLLAFLAPASQTASNLEWSGEDY<br>HQADWGNGHDHL-	Nontranslatable: V, L+V results in frameshift
>3<br>ATGCTGTGGGCTCCAGCCCTCCTTTTAGCTTTCCTGGCTCCTGCCAGTCAGACAGCTTCCAACTTGGAGTGGAG<br>TGGGAGGACTGTCATCAAGCGGACTGGGGAATTGGCCACGATCACTTGTGATGTAGCACAAGAAAGCATCGACT<br>ATGTCCATTGGTACCGATTCCAAGAGGGGATGGTCCCCAAACGTATTCTCTACTACTCTTTGTATTACTCAAAT<br>TCTTATGTAGACCGAGGATTCACAGCAGGGAAATATGATGTTTCTAAAGTTTCAAGCAAGAGCTTCAAATTAGA<br>GGTCCGAAATCTGGAGGAAAGTGATTCCGGTGTGTACAGTTGTGCAGCCTGGGAGTACACGGCTCAGGATGGAT<br>CAAGATTTTTGGACAAGGGACTAAGCTCACAGTAATACCTTCTGGTGGACACCGATCCCCAAAACCCACTATGT<br>TTCTTCCTTCAATTGCTGAAACACATTTTCATAAGGCTGGAACATATCTTTGTCTCCTTGAGGATTTCGTCCCT<br>GACGTTATTAAAGTATATTGGAAAGCAAAGGATGGCAATACGATTCTGGAATCC	V: AAGV03121484.1 (L 100%, V 100%)<br>J: AAGV03121547.1 (100%)<br>C: AAGV03121550.1, AAGV03121548.1 (100%)	V4.1<br>JA<br>C2/3	MLWAPALLLAFLAPASQTASNLEWSGRTVI<br>KRTGELATITCDVAQESIDYVHWYRFQEG<br>MVPKRILYYSLYYSNSYVDRGFTAGKYDV<br>SKVSSKSFKLEVRNLEESDSGVYSCAAWEY<br>TAQDGSRFLDKGLSSQ-	Unproductive VJ rearrangement
>4<br>ATGCTGTGGGCTCCAGCCCTCCTTTTAGCTTTCCTGGCTCCTGCCAGTCAGACAGCTTCCAACTTGGAATGGAG<br>TGGGGAAGACTATCACCAAGCGGACTGGGGAAACGGCCATGATCACTTGTGATATAGCAAAAGAAAACTTCGAC<br>TATCTCCACTGGTACCTGCTCCAAGAGCGGGAGGCCCCCAAACGTGTTCTCTACTACAGTCGGTCTTCCGGAAA<br>GTTTTGGATAGACTCAGGAATCAAACAAGGGAAATATCGTGCTTATAAAGGTTCGGGGGGGAGCTACAGATTTG<br>AGGTCCGAAATCTTGAGGAAAGGGATTCCGGTGTGTATAGCTGTGCAGCCTGGGTCGACGCTAGCTCAGGATGG<br>ATCAAGATTTTTGGACAAGGGACTAAGCTCACAGTAATACCTTCTGGTGGACACCGATCCCCAAAACCCACTAT<br>GTTTCTTCCTTCAATTGCTGAAACACATTTTCATAAGGCTGGAACATATCTTTGTCTCCTTGAGGATTTCGTCC<br>CTGACGTTATTAAAGTATATTGGAAAGCAAAGGATGGCAATACGATTCTGGAATCC	V: AAGV03121496.1 (L 98%, V 99%)<br>J: AAGV03121547.1 (100%)<br>C: AAGV03121550.1, AAGV03121548.1 (100%)	V8.2<br>JA<br>C2/3	MLWAPALLLAFLAPASQTASNLEWSGEDY<br>HQADWGNGHDHL-	Frameshift V region (additional G, PCR artefact?)
>5<br>ATGCTGTGGTCTCTGGCCTTCCTTTTAACTTTCCTGGCTCCTGCCAGTCAGACAGCTTCGAACTTGGAGTGGAG<br>TGGGAGGACTGTCACCAAGGGAACTGGGGAATTGGCCACCATCACTTGTGATATAGAGCTAGGAAACATCCACT<br>ATGTCCACTGGTACCGACTCCAAGAGGGGAAAGGCCCCCAACGTATTCTCTACTACTCTTTGTATTCCTCAAAG<br>TCTTTTGTAGACCGAGGATTCACAGCAGGGAAATATGATGCTTCTAAAGTTTCAAGCAAGAGCTTCAAATTAGA<br>GGTCCGAAATCTGGAGGAAAGTGATTCCGGTGTGTACAGTTGTGCAGCATTCTAAGGAGTGATCAAGATTTTTG<br>GACAAGGGACTAAGCTCACAGTAATTCCTTCTGGTGGACACCGATCCCCAAAACCCACTATGTTTCTTCCTTCA<br>ATTGCTGAAACACATTTTCATAAGGCTGGAACATATCTTTGTCTCCTTGAGGATTTCGTCCCTGACGTTATTAA<br>AGTATATTGGAAAGCAAAGGATGGCATACGATTCTGGAATCCA	V: AAGV03121485.1 (L 100%, V 100%)<br>J: AAGV03121549.1 (98%)<br>C: AAGV03121550.1, AAGV03121548.1 (99%)	V4.2<br>JB<br>C2/3	MLWSLAFLLTFLAPASQTASNLEWSGRTVT<br>KGTGELATITCDIELGNIHYVHWYRLQEGK<br>GPQRILYYSLYSSKSFVDRGFTAGKYDASK<br>VSSKSFKLEVRNLEESDSGVYSCAAF-	Unproductive VJ rearrangement
>6<br>ATGCTGTGGGCTCCAGCCCTCCTTTTAGCTTTCCTGGCTCCTGCCAGTCAGACAGCTTCCAACTTGGAATGGAG<br>TGGGGAAGACTACCACCAAGCGGACTGGGGAAATGGCCACGATCACTTGTGATATAGCGAAAGAAAGCTTTAAC<br>TATCTCCACTGGCACCTGCTCCAAGAGCGTGAGGCCCCCAGACGTATTCTCTACTACGCTCCGTCTTCCGGAAA<br>GTTTTGGATAGACTCGGGAATCAAAGAAGGGAAATATCGTGCTTATAAAGGTTCAGGGAGGAGCTACAGATTCG<br>AGGTCAGGAATCTTGAGGAAAGTGATTCCGGTGTGTATAACTGTGCAGCCTGGGGCCATTAAAGAAGCTAGCTC<br>AGGAGTGATCAAGATTTTTGGACAAGGGACTAAGCTCACAGTAATTCCTTCTGGTGGACACCGATCCCCAAAAC<br>CCACTATGTTTCTTCCTTCAATTGCTGAAACACATTTTCATAATGCTGGGAACATATCTTTGTCTCCTTGAGGA<br>TTTCGTCCCTGACGTTATTAAAGTATATTGGAAAGCAAAGATGGCAATACGATTCTGGAATCC	V: AAGV03121495.1 (L 100%, V 100%)<br>J: AAGV03121549.1 (100%)<br>C: AAGV03121550.1, AAGV03121548.1 (99%)	V4.3<br>JB<br>C2/3	MLWAPALLLAFLAPASQTASNLEWSGEDY<br>HQADWGNGHDHL-	Nontranslatable: V, L+V results in frameshift

>7 (GenBank: MG021119)<br>ATGCTGTGGTCTCTGGCCTTCCTTTTAACTTTCCTGGCTCCTGCCAGTCAGACAGCTTCCAACTTGGAATGGAG<br>TGGGAGGACTGTCACCAAGGGGACTGGGGAATTGGCCACCATCACTTGTGATATAGAGCTAGGAAACATCAACC<br>ATGTCCACTGGTACCGACTCCAAGAGGGGAAAGCCCCCCAACGTATTCTCTACTACTCTTTGTATTCCTCAAAG<br>TCTTTTGTAGACCGAGGATTCACAGCAGGGAAATATGATGCTTCTAAAGTTTCAAGCAAGAGCTTCAAATTAGA<br>GGTCCGAAATCTGGAGGAAAGTGATTCCGGTGTGTACAGTTGTGCAGCCTGGAGCTCAGGATGGATCAAGATTT<br>TTGGACAAGGGACTAAGCTCACAGTAATACCTTCTGGTGGACACCGATCCCCAAAACCCACTATGTTTCTTCCT<br>TCAATTGCTGAAACACATTTTCATAAGGCTGGAACATATCTTTGCCTCCTTGAGGATTTCGTCCCTGACGTTAT<br>TAAAGTATATTGGAAAGCAAAGGATGGCAATACGATTCTGGAATCC	V: AAGV03121485.1 (L 100%, V 98%)<br>J: AAGV03121547.1 (100%)<br>C: AAGV03121550.1, AAGV03121548.1 (99%)	V4.2<br>JA<br>C2/3	MLWSLAFLLTFLAPASQTASNLEWSGRTVT<br>KGTGELATITCDIELGNINHVHWYRLQEGK<br>APQRILYYSLYSSKSFVDRGFTAGKYDASK<br>VSSKSFKLEVRNLEESDSGVYSCAAWSSG<br>WIKIFGQGTKLTVIPSGGHRSPKPTMFLPSIA<br>ETHFHKAGTYLCLLEDFVPDVIKVYWKAK<br>DGNTILES	Translatable
>8 (GenBank: MG021120)<br>ATGCTGTGGGCTCCAACCCTCCTTTTAGCTTTCCTGGCTCCTGCCAGTCAGACAGCTTCCAACTTGGAATGGAG<br>TGGGAAGACTATCACCAAGTTGACTGGGGAAACGGCCACGATCACTTGTGATATAGCAAAAGAAAACTTCGACT<br>ATCTCCACTGGTACCAGCTCCGAGAGCGGGAGGCCCCCAGACGTATTCTCTACTATGCTCCGTCTTCCGCAAAG<br>TTTTGGTTAGACTCGGGAATCAAGCAAGGCAAATACCGCGCTTATAAAATTTCAGGGAGGGGCTACAGATTCGA<br>GGTCCGAAATCTGGAGGAAAGTGACTCCAGTGTGTACAGTTGTGCAGCCTGGGAGGGGGAAGCTAGCTCAGGAG<br>TGATCAAGATTTTTGGACAAGGGACTAAGCTCACAGTAATTCCTTCTGGTGGACACCGATCCCCAAAACCCACT<br>ATGTTTCTTCCTTCAATTGCTGAAACACATTTTCATAAGGCTGGAACATATCTTTGTCTCCTTGAGGATTTCGT<br>CCCTGACGTTATTAAAGTATATTGGAAAGCAAAGGATGGCAATACGATTCTGGAATCCA	V: AAGV03121496.1 (L 100%, V 90%)<br>J: AAGV03121549.1 (100%)<br>C: AAGV03121550.1, AAGV03121548.1 (100%)	V8.4<br>JB<br>C2/3	MLWAPTLLLAFLAPASQTASNLEWSGKTIT<br>KLTGETATITCDIAKENFDYLHWYQLRERE<br>APRRILYYAPSSAKFWLDSGIKQGKYRAYK<br>ISGRGYRFEVRNLEESDSSVYSCAAWEGEA<br>SSGVIKIFGQGTKLTVIPSGGHRSPKPTMFLP<br>SIAETHFHKAGTYLCLLEDFVPDVIKVYWK<br>AKDGNTILES	Translatable
>9<br>ATGCTGTGGTCTCTGGCCTTCCTTTTAACTTTCCTGGCTCATTCCAGTCAGACAGCTTCGAACTTGGAGTGGAG<br>TGGGAGGACTGTCACCAAGGGAACTGGGGAATTGGCCACCATCACTTGTGATATAGAGCTAGGAAACATCCACT<br>ATGTCCACTGGTACCGACTCCAAGAGGGGAAAGGCCCCCAACGTATTCTCTACTACTCTTTGTATTCCTCAAAG<br>TCTTTTGTAGACCGAGGATTCACAGCAGGGAAATATGATGCTTCTAAAGTTTCAAGCAAGAGCTTCAAATTAGA<br>GGTCCGAAATCTGGAGGAAAGTGATTCCGGTGTGTACAGTTGTGCAGCCTGGCTAGCTCAGGAGTGATCAAGAT<br>TTTTGGACAAGGGACTAAGCTCACAGTAATTCCTTCTGGTGGACACCGATCCCCAAAACCCACTATGTTTCTTC<br>CTTCAATTGCTGAAACACATTTTCATAAGGCTGGAACATATCTTTGTCTCCTTGAGGATTTCGTCCCTGACGTT<br>ATTAAAGTATATTGGAAAGCAAAGGATGGCAATACGATTCTGGAATCC	V: AAGV03121485.1 (L 100%, V 99%)<br>J: AAGV03121549.1 (100%)<br>C: AAGV03121550.1, AAGV03121548.1 (100%)	V4.2<br>JB<br>C2/3	MLWSLAFLLTFLAHSSQTASNLEWSGRTVT<br>KGTGELATITCDIELGNIHYVHWYRLQEGK<br>GPQRILYYSLYSSKSFVDRGFTAGKYDASK<br>VSSKSFKLEVRNLEESDSGVYSCAAWLAQE<br>-	Unproductive VJ rearrangement
>10<br>ATGCTGTGGGCTCCAGCCCTCCTTTTAGCTTTCCTGGCTCCTGCCAGTCAGACAGCTTCCAACTTGGAATGGAG<br>TGGGGAAGACTACCACCAAGCGGACTGGGGAAATGGCCACGATCACTTGTGATATAGCGAAAGAAAGCTTTAAC<br>TATCTCCACTGGCACCTGCTCCAAGAGCGTGAGGCCCCCAGACGTATTCTCTACTACGCTCCGTCTTCCGGAAA<br>GTTTTGGATAGACTCGGGAATCAAAGAAGGGAAATATCGTGCTTATAAAGGTTCAGGGAGGAGCTACAGATTCG<br>AGGTCAGGAATCTTGAGGAAAGTGATTCCGGTGTGTATAACTGTGCAGCCTGGGCTAGCTCAGGAGTGATCAAG<br>ATTTTTGGACAAGGGACTAAGCTCACAGTAATTCCTTCTGGTGGACACCGATCCCCAAAACCCACTATGTTTCT<br>TCCTTCAATTGCTGAAACACATTTTCATAAGCTGGAACATATCTTTGTCTCCTTGAGGATTTCGTCCCTGACGT<br>TATTAAAGTAATATTGGAAAGCAAAGGATGGCAATACGATTCTGGAATCC	V: AAGV03121495.1 (L 100%, V 100%)<br>J: AAGV03121549.1 (98%)<br>C: AAGV03121550.1, AAGV03121548.1 (99%)	V4.3<br>JB<br>C2/3	MLWAPALLLAFLAPASQTASNLEWSGEDY<br>HQADWGNGHDHL-	Nontranslatable: V, L+V results in frameshift
>11<br>ATGCTGTGGGCTCCAGCCCTCCTTTTAGCTTTCCTGGCTCCTGCCAGTCAGACAGCTTCCAACTTGGAATGGAG<br>TGGGGAAGACTACCACCAAGCGGACTGGGGAAATGGCCACGATCACTTGTGATATAGCGAAAGAAAGCTTTAAC<br>TATCTCCACTGGCACCTGCTCCAAGAGCGTGAGGCCCCCAGACGTATTCTCTACTACGCTCCGTCTTCCGGAAA<br>GTTTTGGATAGACTCGGGAATCAAAGAAGGGAAATATCGTGCTTATAAAGGTTCAGGGAGGAGCTACAGATTCG<br>AGGTCAGGAATCTTGAGGAAAGTGATTCCGGTGTGTATAACTGTGCAGCCTGGGTGTGAACTCAGGATGGATCA<br>AGATTTTTGGACAAGGGACTAAGCTCACGGTAATACCTTCTGGTGGACACCGATCCCCAAAACCCACTATGTTT<br>CTTCCTTCAATTGCTGAAACACATTTTCATAAGGCTGGAACATATCTTTGTCTCCTTGAGGATTTCGTCCCTGA<br>CGTTATTAAAGTATATTGGAAAGCAAAGGATGGCAATACGATTCTGGAATCC	V: AAGV03121495.1 (L 100%, V 100%)<br>J: AAGV03121547.1 (98%)<br>C: AAGV03121550.1, AAGV03121548.1 (100%)	V4.3<br>JA<br>C2/3	MLWAPALLLAFLAPASQTASNLEWSGEDY<br>HQADWGNGHDHL-	Nontranslatable: V, L+V results in frameshift
>12<br>ATGCTGTGGGCTCCAGCCCTCCTTTTAGCTTTCCTGGCTCCTGCCAGTCAGACAGCTTCCAACTTGGAGTGGAG<br>TGGGAGGACTGTCATCAAGCGGACTGGGGAATTGGCCACGATCACTTGTGATGTAGCACAAGAAAGCATCGACT<br>ATGTCCATTGGTACCGATTCCAAGAGGGGATGGTCCCCAAACGTATTCTCTACTACTCTTTGTATTACTCAAAT	V: AAGV03121484.1 (L 100%, V 99%)<br>J: AAGV03121547.1 (98%)	V4.1<br>JA<br>C2/3	MLWAPALLLAFLAPASQTASNLEWSGRTVI<br>KRTGELATITCDVAQESIDYVHWYRFQEG<br>MVPKRILYYSLYYSNSYVDRGFTAGKYDV	Unproductive VJ rearrangement

TCTTATGTAGACCGAGGATTCACAGCAGGGAAATATGATGTTTCTAAAGTTTCAAGCAAGAGCTTCAAGTTAGA<br>GGTCCGAAACCTGGAGGAAAGTGATTCCGGTGTGTACAGTTGTGCAGCCTGGGAGGATGGATCAAGATTTTTGG<br>ACAAGGGACTAAGCTCACAGTAATACCTTCTGGTGGACACCGATCCCCAAAACCCACTATGTTTCTTCCTTCAA<br>TTGCTGAAACACATTTTCATAAGGCTGGAACATATCTTTGTCTCCTTGAGGATTTCGTCCCTGACGTTATTAAA<br>GTATATTGGAAAGCAAAGGATGGCAATACGATTCTGGAATCC	C: AAGV03121550.1,<br>AAGV03121548.1 (100%)		SKVSSKSFKLEVRNLEESDSGVYSCAAWED<br>GSRFLDKGLSSQ-	
>13 (GenBank: MG021121)<br>ATGCTGCTCCCGCTACAGGTCGTCGCGGTGGCTTCTCTCTGGGCGTATTCAAGTGGCAACAGTCTCATAACGCA<br>GCTCGCGGCATCCATCACCAAGAGAAAAGGAAATACGGCCTTTTTTGAATGCCAAGTGGAAACAAGTGTTTTAA<br>AGAAAAACAGGTTTATACACTGGTACCAGCAGAAGCCAGGCCGGCCTCTGGAACGAATTCTGTATATTTCCTCA<br>AATGAAAATATTTTCTATGAAAAAGGCATCAGCGAGGAAAGATTCGAGGCCAGGAAGCGGCAGGACGGCTCGGC<br>GGCGAGCCTCAGAGTGCACCGCGTGGCGGAGGCGGACGCGGGCACGTACTACTGCGCCTGCTGGGTCACCGGGG<br>AGAGCGTCGAAGTCTTTGGTCCTGGCACAAAGCTCGTTGTTCCAGATAACACCCCGAAGGGAGACCTATCCCCC<br>AAACCCACTGTGTTTCTTCCTTCAATTGCTGAAACACATCTTCATAAGGCTGGAACATATCTCTGTCTTCTTGA<br>GGATTTCTTCCCTGACGCTATTAAAGTATATTGGAAAGCAAAGGATGGCAATACGATTCTGGAATCC	V: AAGV03121542.1 (L 100%,<br>V 100%)<br>J: AAGV03121543.1 (94%)<br>C1: AAGV03121543.1 (100%)	mV6<br>JC<br>C1	MLLPLQVVAVASLWAYSSGNSLITQLAASI<br>TKRKGNTAFFECQVETSVLKKNRFIHWYQ<br>QKPGRPLERILYISSNENIFYEKGISEERFEA<br>RKRQDGSAASLRVHRVAEADAGTYYCAC<br>WVTGESVEVFGPGTKLVVPDNTPKGDLSP<br>KPTVFLPSIAETHLHKAGTYLCLLEDFFPDA<br>IKVYWKAKDGNTILES	Translatable
>14 (GenBank: MG021122)<br>ATGCTGTGGGCTCCAGCCCTCCTTTTAGCTTTCCTGGCTCCTGCCAGTCAGACAGCTTCCAACTTGGAGTGGAG<br>TGGGAGGACTGTCATCAAGCGGACTGGGGAATTGGCCACGATCACTTGTGATGTAGCACAAGAAAGCATCGACT<br>ATGTCCATTGGTACCGATTCCAAGAGGGGATGGTCCCCAAACGTATTCTCTACTACTCTTTGTATTACTCAAAT<br>TCTTATGTAGACCGAGGATTCACAGCAGGGAAATATGATGTTTCTAAAGTTTCAAGCAAGAGCTTCAAATTAGA<br>GGTCCGAAATCTGGAGGAAAGTGATTCCGGTGTGTACAGTTGTGCAGCCTGGGGGGAATCAGGAGTGATCAAGA<br>TTTTTGGACAAGGGACTAAGCTCACAGTAATTCCTTCTGGTGGACACCGATCCCCAAAACCCACTATGTTTCTT<br>CCTTCAATTGCTGAAACACATTTTCATAAGGCTGGAACATATCTTTGTCTCCTTGAGGATTTCGTCCCTGACGT<br>TATTAAAGTATATTGGAAAGCAAAGGATGGCAATACGATTCTGGAATCC	V: AAGV03121484.1 (L 100%,<br>100%)<br>J: AAGV03121549.1 (100%)<br>C: AAGV03121550.1,<br>AAGV03121548.1 (100%)	V4.1<br>JB<br>C2/3	MLWAPALLLAFLAPASQTASNLEWSGRTVI<br>KRTGELATITCDVAQESIDYVHWYRFQEG<br>MVPKRILYYSLYYSNSYVDRGFTAGKYDV<br>SKVSSKSFKLEVRNLEESDSGVYSCAAWGE<br>SGVIKIFGQGTKLTVIPSGGHRSPKPTMFLPS<br>IAETHFHKAGTYLCLLEDFVPDVIKVYWKA<br>KDGNTILES	Translatable
>15<br>ATGCTGTGGGCTCCAGCCCTCCTTTTAGCTTTCCTGGCTCCTGCCAGTCAGGCAGCTTCCAACTTGGAATGGAG<br>TGGGGAAGACTACCACCAAGCGGACTGGGGAAATGGCCACGATCACTTGTGATATAGCGAAAGAAAGCTTTAAC<br>TATCTCCACTGGCACCTGCTCCAAGAGCGTGAGGCCCCCAGACGTATTCTCTACTACGCTCCGTCTTCCGGAAA<br>GTTTTGGATAGACTCGGGAATCAAAGAAGGGAAATATCGTGCTTATAAAGGTTCAGGGAGGAGCTACAGATTCG<br>AGGTCAGGAATCTTGAGGAAAGTGATTCCGGTGTGTATAACTGTGCAGCCTGGGAGAGTAGCTCAGGAGTGATC<br>AAGATTTTTGGACAAGGGACTAAGCTCACAGTAATTCCTTCTGGTGGACACCGATCCCCAAAACCCACTATGTT<br>TCTTCCTTCAATTGCTGAAACACATTTTCATAAGGCTGGAACATATCTTTGTCTCCTTGAGGATTTCGTCCCTG<br>ACGTTATTAAAGTATATTGGAAAGCAAAGGATGGCAATACGATTCTGGAATCC	V: AAGV03121495.1 (L 100%,<br>V 99%)<br>J: AAGV03121549.1 (100%)<br>C: AAGV03121550.1,<br>AAGV03121548.1 (100%)	V4.3<br>JB<br>C2/3	MLWAPALLLAFLAPASQAASNLEWSGEDY<br>HQADWGNGHDHL-	Nontranslatable:<br>V, L+V results in<br>frameshift
>16<br>ATGCTGTGGGCTCCAGCCCTCCTTTTAGCTTTCCTGGCTCCTGCCAGTCAGACAGCTTCCAACTTGGAATGGAG<br>TGGGGAAGACTACCACCAAGCGGACTGGGGAAATGGCCACGATCACTTGTGATATAGCGAAAGAAAGCTTTAAC<br>TATCTCCACTGGCACCTGCTCCAAGAGCGTGAGGCCCCCAGACGTATTCTCTACTACGCTCCGTCTTCCGGAAA<br>GTTTTGGATAGACTCGGGAATCAAAGAAGGGAAATATCGTGCTTATAAAGGTTCAGGGAGGAGCTACAGATTCG<br>AGGTCAGGAATCTTGAGGAAAGTGATTCCGGTGTGTATAACTGTGCAGCCTGGCTCAGGATGGATCAAGATTTA<br>TGGACAAGGGACTAAGCTCACAGTAATACCTTCTGGTGGACACCGATCCCCAAAACCCACTATGTTTCTTCCTT<br>CAATTGCTGAAACACATTTTCATAAGGCTGGAACATATCTTTGTCTCCTTGAGGATTTCGTCCCTGACGTTATT<br>AAAGTATATTGGAAAGCAAAGGATGGCAATACGATTCTGGAATCCA	V: AAGV03121495.1 (L 100%,<br>V 99%)<br>J: AAGV03121547.1 (97%)<br>C: AAGV03121550.1,<br>AAGV03121548.1 (100%)	V4.3<br>JA<br>C2/3	MLWAPALLLAFLAPASQTASNLEWSGEDY<br>HQADWGNGHDHL-	Nontranslatable:<br>V, L+V results in<br>frameshift
>17 (GenBank: MG021123)<br>ATGCTGTGGGCTCCAGCCCTCCTTTTAGCTTTCCTGGCTCCTGCCAGCCAGACAACTTCCAGCTTGGAGTGGAG<br>TGGGAGGACTGTCACCAAGCAGACTGGGGAATTGGCCACCATCACTTGTGATGTAGCACAAGAATACATCAACT<br>ATGTCCACTGGTACCGATTCCAAGAGGGGATGGTCCCCAAACGTATTCTCTACTACTCTTTGTATTCCTCAAAG<br>CCTTATGTAGACTCAGGATTCACAACAGGGAAATATGGTGCTTCTAAAGTTTCAAGTAAGAGCTACAAGTTAGA<br>GGTCCAAAATCTGGAGGAAAGTGATTCCGGTGTGTACAGTTGTGCAGCCTGGCGACTGAGCTCAGGAGTGATCA<br>AGATTTTTGGACAAGGGACTAAGCTCACAGTAATTCCTTCTGGTGGACACCGATCCCCAAAACCCACTATGTTT	V: AAGV03121491.1 (L 100%,<br>V 100%)<br>J: AAGV03121549.1 (100%)<br>C: AAGV03121550.1,<br>AAGV03121548.1 (100%)	V2<br>JB<br>C2/3	MLWAPALLLAFLAPASQTTSSLEWSGRTVT<br>KQTGELATITCDVAQEYINYVHWYRFQEG<br>MVPKRILYYSLYSSKPYVDSGFTTGKYGAS<br>KVSSKSYKLEVQNLEESDSGVYSCAAWRL<br>SSGVIKIFGQGTKLTVIPSGGHRSPKPTMFLP<br>SIAETHFHKAGTYLCLLEDFVPDVIKVYWK<br>AKDGNTILES	Translatable

CTTCCTTCAATTGCTGAAACACATTTTCATAAGGCTGGAACATATCTTTGTCTCCTTGAGGATTTCGTCCCTGA<br>CGTTATTAAAGTATATTGGAAAGCAAAGGATGGCAATACGATTCTGGAATCC				
>18<br>ATGCTGTGGGCTCCAGCGCTCCTTTTAGCTTTCCTGGCTCCTGCCAGTCAGACAGCTTCCAACTTGGAGTGGAG<br>TGGGAGGACTGTCAGCAAGCAGACTGGGGAATTGGCCATGATCACTTGTGATGTAGCACAAGAAAACATTGACT<br>ATGTCCACTGGTACCGACTCCAAGAGGGGGAGGCCCCCAAACGTATTCTCCACTACTCTTCATCTTCCACAAAT<br>TTCTGGACAGACTCGGGAATCAAACGAGCGAAATATGGTGCTTTTAAAGCTTCAGGGAGGAGCTACAAATTAGA<br>GGTCCGAAATGTGGAGGAAAGTGATTCCGGTGTGTACAGTTGTGCAGCCTGGGAGCGTCCTAGCTCAGGAGTGA<br>TCAAGATTTTTGGACAAGGGACTAAGCTCACAGTAATTCCTTCTGGTGGACACCGATCCCCAAAACCCACTATG<br>TTTCTTCCTTCAATTGCTGAAACACATTTTCATAAGGCTGGAACATATCTTTGTCTCCTTGAGGATTTCGTCCC<br>TGACGTTATTAAAGTATATTGGAAAGCAAAGGATGGCAATACGATTCTGGAATCC	V: AAGV03121492.1 (L 100%, V 100%)<br>J: AAGV03121549.1 (96%)<br>C: AAGV03121550.1, AAGV03121548.1 (100%)	V8.3<br>JB<br>C2/3	MLWAPALLLAFLAPASQTASNLEWSGRTV<br>SKQTGELAMITCDVAQENIDYVHWYRLQE<br>GEAPKRILHYSSSSTNFWTDSGIKRAKYGA<br>FKASGRSYKLEVRNVEESDSGVYSCAAWE<br>RPSSGVIKILDKGLSSQ-	Unproductive VJ rearrangement
>19 (GenBank: MG021124)<br>ATGCTGTGGGCTCCAGCCCTCCTTTTAGCTTTCCTGGCTCCTGCTTCCAACTTGGAATGGAGTGGGAAGACTAT<br>CACCAAGTTGACTGGGGAAACGGCCACGATCACTTGTGATATAGCAAAAGAAAACTTCGACTATCTCCACTGGT<br>ACCAGCTCCGAGAGCGGGAGGCCCCCAGACGTATTCCCTACTATGCTCCGTCTTCCGCAAAGTTTTGGTTAGAC<br>TCGGGAATCAAGCAAGGCAAATACCGCGCTTATAAAATTTCAGGGAGGGGCTACAGATTCGAGGTCCGAAATCT<br>GGAGGAAAGTGACTCCAGTGTGTACAGTTGTGCAGGGCCCGGAGTGATCAAGATTTTTGGACAAGGGACTAAGC<br>TCACAGTAATTCCTTCTGGTGGACACCGATCCCCAAAACCCACTATGTTTCTTCCTTCAATTGCTGAAACACAT<br>TTTCATAAAGCTGGAACATATCTTTGTCTCCTTGAGGATTTCGTCCCTGACGTTATTAAAGTATATTGGAAAGC<br>AAAGGATGGCAATACGATTCTGGAATCCA	V: AAGV03121496.1 (L98%, V 90%)<br>J: AAGV03121549.1 (100%)<br>C: AAGV03121550.1, AAGV03121548.1 (99%)	V8.4<br>JB<br>C2/3	MLWAPALLLAFLAPASNLEWSGKTITKLTG<br>ETATITCDIAKENFDYLHWYQLREREAPRRI<br>PYYAPSSAKFWLDSGIKQGKYRAYKISGRG<br>YRFEVRNLEESDSSVYSCAGPGVIKIFGQGT<br>KLTVIPSGGHRSPKPTMFLPSIAETHFHKAG<br>TYLCLLEDFVPDVIKVYWKAKDGNTILES	Translatable
>20<br>ATGCTGTGGGCTCCAGCCCTCCTTTTAGCTTTCCTGGCTCCTGCTTCCAACTTGGAATGGAGTGGGAAGACTAT<br>CACCAAGTTGACTGGGGAAACGGCCACGATCACTTGTGATATAGCAAAAGAAAACTTCGACTATCTCCACTGGT<br>ACCAGCTCCGAGAGCGGGAGGCCCCCAGACGTATTCTCTACTATGCTCCGTCTTCCGCAAAGTTTTGGTTAGAC<br>TCGGGAATCAAGCAAGGCAAATACCGCGCTTATAAAATTTCAGGGAGGGGCTACAGATTCGAGGTCCGAAATCT<br>GGAGGAAAGTGACTCCAGTGTGTACAGTTGTGCAGCCTGGATAGCTCAGGAGTGATCAAGATTTTTGGACAAGG<br>GACTAAGCTCACAGTAATTCCTTCTGGTGGACACCGATCCCCAAAACCCACTATGTTTCTTCCTTCAATTGCTG<br>AAACACATTTTCATAAGGCTGGAACATATCTTTGTCTCCTTGAGGATTCCGTCCCTGACGTTATTAAAGTATAT<br>TGGAAAGCAAAGGATGGCAATACGATTCTGGAATCC	V: AAGV03121496.1 (L98%, V 90%)<br>J: AAGV03121549.1 (100%)<br>C: AAGV03121550.1, AAGV03121548.1 (99%)	V8.4<br>JB<br>C2/3	MLWAPALLLAFLAPASNLEWSGKTITKLTG<br>ETATITCDIAKENFDYLHWYQLREREAPRRI<br>LYYAPSSAKFWLDSGIKQGKYRAYKISGRG<br>YRFEVRNLEESDSSVYSCAAWIAQE-	Unproductive VJ rearrangement
>21<br>ATGCTGTGGTCTCTGGCCTTCCTTTTAACTTTCCTGGCTCCTGCCAGTCAGACAGCTTCCAACTTGGAATGGAG<br>TGGGAGGACTGTCACCAAGGGGACTGGGGAATTGGCCACCATCACTTGTGATATAGAGCTAGGAAACATCAACC<br>ATGTCCACTGGTACCGACTCCAAGAGGGGAAAGCCCCCCAACGTATTCTCTACTACTCTTTGTATTCCTCAAAG<br>TCTTTTGTAGACCGAGGATTCACAGCAGGGAAATATGATGCTTCTAAAGTTTCAAGCAAGAGCTTCAAATTAGA<br>GGTCCGAAATCTGGAGGAAAGTGATTCCGGTGTGTACAGTTGTGCAGCCTGGGAGTGGTCAGCTCAGGAGTGAT<br>CAAGATTTTTGGACAAGGGACTAAGCTCACAGTAATTCCTTCTGGTGGACACCGATCCCCAAAACCCACTATGT<br>TTCTTCCTTCAATTGCTGAAAC	V: AAGV03121485.1 (L 100%, V 98%)<br>J: AAGV03121549.1 (100%)<br>C: AAGV03121550.1, AAGV03121548.1 (100%)	V4.2<br>JB<br>C2/3	MLWSLAFLLTFLAPASQTASNLEWSGRTVT<br>KGTGELATITCDIELGNINHVHWYRLQEGK<br>APQRILYYSLYSSKSFVDRGFTAGKYDASK<br>VSSKSFKLEVRNLEESDSGVYSCAAWEWS<br>AQE-	Unproductive VJ rearrangement
>22 (GenBank: MG021125)<br>ATGCTGTGGGCTCCAGCCCTCCTTTTAGCTTTCCTGGCTCCTGCCAGCCAGACAACTTCCAGCTTGGAGTGGAG<br>TGGGAGGACTGTCACCAAGCAGACTGGGGAATTGGCCACCATCACTTGTGATGTAGCACAAGAATACATCAACT<br>ATGTCCACTGGTACCGATTCCAAGAGGGGATGGTCCCCAAACGTATTCTCTACTACTCTTTGTATTCCTCAAAG<br>CCTTATGTAGACTCAGGATTCACAACAGGGAAATATGGTGCTTCTAAAGTTTCAAGTAAGAGCTACAAGTTAGA<br>GGTCCAAAATCTGGAGGAAAGTGATTCCGGTGTGTACAGTTGTGCAGCCTGGCGAATCGGTAGCTCAGGAGTGA<br>TCAAGATTTTTGGACAAGGGACTGAGCTCACAGTAATTCCTTCTGGTGGACACCGATCCCCAAAACCCACTATG<br>TTTCTTCCTTCAATTGCTGAAACACATTTTCATAAGGCTGGAACATATCTTTGTCTCCTTGAGGATTTCGTCCC<br>TGACGTTATTAAAGTATATTGGAAAGCAAAGGATGGCAATACGATTCTGGAATCC	V: AAGV03121491.1 (L 100%, V 100%)<br>J: AAGV03121549.1 (98%)<br>C: AAGV03121550.1, AAGV03121548.1 (100%)	V2<br>JB<br>C2/3	MLWAPALLLAFLAPASQTTSSLEWSGRTVT<br>KQTGELATITCDVAQEYINYVHWYRFQEG<br>MVPKRILYYSLYSSKPYVDSGFTTGKYGAS<br>KVSSKSYKLEVQNLEESDSGVYSCAAWRIG<br>SSGVIKIFGQGTELTVIPSGGHRSPKPTMFLP<br>SIAETHFHKAGTYLCLLEDFVPDVIKVYWK<br>AKDGNTILES	Translatable
>23<br>ATGCTGTGGGCTCCAGCGCTCCTTTTAGCTTTCCTGGCTCCTGCCAGTCAGACAGCTTCCAACTTGGAGTGGAG<br>TGGGAGGACTGTCAGCAAGCAGACTGGGGAATTGGCCATGATCACTTGTGATGTAGCACAAGAAAACATTGACT	V: AAGV03121492.1 (L100%, V 100%)<br>J: AAGV03121549.1 (100%)	V8.3<br>JB<br>C2/3	MLWAPALLLAFLAPASQTASNLEWSGRTV<br>SKQTGELAMITCDVAQENIDYVHWYRLQE<br>GEAPKRILHYSSSSTNFWTDSGIKRAKYGA	Unproductive VJ rearrangement

ATGTCCACTGGTACCGACTCCAAGAGGGGGAGGCCCCCAAACGTATTCTCCACTACTCTTCATCTTCCACAAAT<br>TTCTGGACAGACTCGGGAATCAAACGAGCGAAATATGGTGCTTTTAAAGCTTCAGGGAGGAGCTACAAATTAGA<br>GGTCCGAAATGTGGAGGAAAGTGATTCCGGTGTGTACAGTTGTGCAGCCTGGGGGCTCAGGAGTGATCAAGATT<br>TTTGGACAAGGGACTAAGCTCACAGTAATTCCTTCTGGTGGACACCGATCCCCAAAACCCACTATGTTTCTTCC<br>TTCAATTGCTGAAACACATTTTCATAAGGCTGGAACATATCTTTGTCTCCTTGAGGATTTCGTCCCTGACGTTA<br>TTAAAGTATATTGGAAAGCAAAGGATGGCAATACGATTCTGGAATCC	C: AAGV03121550.1,<br>AAGV03121548.1 (100%)		FKASGRSYKLEVRNVEESDSGVYSCAAWG<br>LRSDQDFWTRD-	
>24 (GenBank: MG021126<br>ATGCTGTGGGCTCCAGCCCTCCTTTTAGCTTTCCTGACTCCCTCCAGTCAGACAGCTTTCAACCTGGAATGGAG<br>TGGGAAGTCTTTCACGAAGCAGACTGGGAAATTTGCTACAATCCCTTGTGATATAGTGAAAGAAAACATCGACT<br>ATGTCCACTGGTACCAACACCAAGAAGGGGAGGTCCCCAAACGAATTCTCTACTACTCTTTTCTTTCCTCAAAG<br>TTTTCAGTAGACTCAGGAATCAGTCAAGGGAAATATCATGCTTCTGAAAGTACAGGGAGAAGCTGCAAATTTGT<br>AGTTCGAAATTTAGAGAAAAGTGATTCTGGCGTGTATTATTGTGCGGCCTGGGACATGGGCGAGAGCGTCAAAG<br>TCTTTGGTCCTGGCACAAAGCTCGTTGTTCCAGATAACACCCCGAAGGGAGACCTATCCCCCAAACCCACTGTG<br>TTTCTTCCTTCAATTGCTGAAACACATCTTCATAAGGCTGGAACATATCTCTGTCTTCTTGAGGATTTCTTCCC<br>TGACGCTATTAAAGTATATTGGAAAGCAAAGGATGGCAATACGATTCTGGAATCC	V: AAGV03121497.1 (L 100%,<br>V 100%)<br>J: AAGV03121543.1 (98%)<br>C: AAGV03121543.1 (100%)	V8.1<br>JC<br>C1	MLWAPALLLAFLTPSSQTAFNLEWSGKSFT<br>KQTGKFATIPCDIVKENIDYVHWYQHQEGE<br>VPKRILYYSFLSSKFSVDSGISQGKYHASES<br>TGRSCKFVVRNLEKSDSGVYYCAAWDMG<br>ESVKVFGPGTKLVVPDNTPKGDLSPKPTVF<br>LPSIAETHLHKAGTYLCLLEDFFPDAIKVY<br>WKAKDGNTILES	Translatable

References

Nguyen, K., Li, J., Puthenveetil, R., Lin, X., Poe, M.M., Hsiao, C.C., et al. (2017). The butyrophilin 3A1 intracellular domain undergoes a conformational change involving the juxtamembrane region. *FASEB J* 31(11)**,** 4697-4706. doi: 10.1096/fj.201601370RR.

Sandstrom, A., Peigne, C.M., Leger, A., Crooks, J.E., Konczak, F., Gesnel, M.C., et al. (2014). The intracellular B30.2 domain of butyrophilin 3A1 binds phosphoantigens to mediate activation of human Vgamma9Vdelta2 T cells. *Immunity* 40(4)**,** 490-500. doi: 10.1016/j.immuni.2014.03.003.

Vavassori, S., Kumar, A., Wan, G.S., Ramanjaneyulu, G.S., Cavallari, M., El Daker, S., et al. (2013). Butyrophilin 3A1 binds phosphorylated antigens and stimulates human gammadelta T cells. *Nat Immunol* 14(9)**,** 908-916. doi: 10.1038/ni.2665.
